# Photocatalytically Activated Cu‐N_1_S_3_ Single‐Atom Nanozyme: Enhancing Enzyme Activities and Antibacterial Synergy for Highly Efficient Fruit Preservation

**DOI:** 10.1002/advs.202515542

**Published:** 2025-12-12

**Authors:** Chuanlong Men, Chenchen Wu, Lei Wang, Shengjie Gao, Yu Mao, Wei Liu, Changhong Liu, Lei Zheng

**Affiliations:** ^1^ Engineering Research Center of Bio‐Process Ministry of Education School of Food and Biological Engineering Hefei University of Technology Hefei 230009 China

**Keywords:** coating film, Cu single‐atom nanozymes, photocatalytic coordination, synergistic antibacterial, α‐lipoic acid

## Abstract

Postharvest preservation urgently demands innovative solutions bridging atomic precision with practical scalability. Here, a distinctive photocatalysis‐driven self‐assembly strategy is presented that fundamentally diverged from conventional high‐temperature syntheses by enabling precise single‐atom coordination under ambient conditions. This approach, utilizing α‐lipoic acid (α‐LA) as coordination ligand, achieved the mild assembly of S‐coordinated Cu single‐atom nanozymes (Cu/CNS) while significantly enhancing their enzymatic activity. The resulting material demonstrated unprecedented multi‐enzyme mimetic activities (catalase‐, oxidase‐, and glutathione oxidase‐like) with catalytic efficiency surpassing conventional nanozymes by orders of magnitude. The Cu/CNS exhibits near‐perfect antimicrobial efficacy against *Escherichia coli* (*E. coli*), *Staphylococcus aureus* (*S. aureus*), and *Botrytis cinerea* (*B. cinerea*) through synergistic mechanisms. When integrated into chitosan‐gelatin films (Cu/CNS@CS‐Gel), it forms active packaging with pH‐responsive behavior, exceptional barrier properties, and mechanical strength. Crucially, the synthesis is simple, scalable, and environmentally adaptable. Using strawberries and kiwifruits as representative examples, Cu/CNS@CS‐Gel more than doubled the shelf life while efficiently maintaining nutritional quality. Beyond food packaging, this coordination chemistry platform is generalizable to other metal‐ligand systems, offering a versatile toolbox for sustainable agriculture. By bridging atomic‐level design with practical feasibility, the work advances sustainable nanozyme implementation in food systems.

## Graphical abstract

This study presents S‐coordinated Cu single‐atom nanozymes (Cu/CNS) fabricated via mild photocatalytic self‐assembly strategy. The nanozyme exhibits exceptional multi‐enzyme activity and near‐perfect antimicrobial efficacy. Incorporated into chitosan‐gelatin film, it enables pH‐responsive, broad‐spectrum fruit preservation, more than doubling the shelf‐life of strawberries and kiwifruits while maintaining their nutritional quality, offering a scalable and sustainable solution for food packaging.

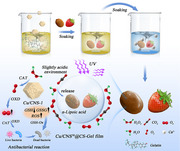

## 1 Introduction

Postharvest fruit loss poses a significant global challenge because it affects food security, resource sustainability, and economic efficiency. According to the Food and Agriculture Organization (FAO) of the United Nations, ≈1.3 billion tons of fruits and vegetables is lost annually; postharvest losses account for 30–40% of this total, resulting in economic losses of ≈$1 trillion.^[^ ^1^ ^]^ These losses are primarily caused by physiological metabolic activities, environmental fluctuations, pathogen infections, and mechanical damage during storage and transportation, leading to quality deterioration such as dehydration, softening, and decay.^[^ ^2^ ^]^ Traditional preservation methods, including cold storage, controlled‐atmosphere storage, and chemical treatments, are often limited by issues such as technical complexity, high costs, and insufficient efficacy.^[^ ^3^ ^]^ Therefore, the development of safe, efficient, and intelligent preservation technologies is crucial to maintain fruit freshness and quality, reduce food waste, and ensure food safety. Advanced research in this field holds significant theoretical and strategic importance for increasing market competitiveness and addressing global food security challenges.

Natural bioactive compounds, such as α‐lipoic acid (α‐LA), have sparked great interest as alternatives to chemical preservatives for fruit storage and preservation.^[^ ^4^ ^]^ α‐LA, a multifunctional natural compound, exhibits antioxidant, anti‐inflammatory, and antimicrobial properties, effectively regulating plant growth and development by scavenging reactive oxygen species (ROS), modulating circadian rhythms, and delaying fruit ripening and senescence.^[^ ^5^ ^]^ Studies, including our own, have shown that α‐LA treatment enhances the storage life and quality of postharvest fruits, such as litchi,^[^ ^6^ ^]^ strawberry,^[^ ^7^ ^]^ and pear,^[^ ^8^ ^]^ by improving their antioxidant capacity, energy metabolism, and phenolic and carbohydrate metabolism. In addition to these well‐documented benefits, α‐LA may offer particularly unique advantages for constructing functional preservation materials. Its distinctive disulfide bond holds the potential to enable efficient photocatalytic cleavage, generating thiol groups in situ that facilitate precise coordination with metal active sites and promote the formation of stable composite structures. This promising combination of photocatalytic responsiveness and intrinsic bioactivity could position α‐LA as a highly suitable ligand for developing advanced preservation systems that extend beyond conventional applications. However, the practical application of natural preservatives like α‐LA is limited by challenges such as uncontrolled release and insufficient environmental responsiveness. Therefore, the development of advanced preservation materials with precise loading and intelligent release capabilities remains both a critical challenge and key direction for innovations in postharvest fruit preservation technologies.

Single‐atom nanozymes, which are characterized by atomically dispersed active sites, can achieve nearly 100% atomic utilization and exhibit excellent catalytic performance, high stability, and tunable electronic properties.^[^ ^9^ ^]^ These features have enabled their widespread use in catalysis, medicine, and food detection.^[^ ^10^, ^11^, ^12^, ^13^, ^14^, ^15^ ^]^ Notably, the antimicrobial and antioxidant properties of nanozymes support their potential use as advanced food preservation materials.^[^ ^16^, ^17^, ^18^, ^19^, ^20^, ^21^ ^]^ The atomically dispersed structure of single‐atom nanozymes ensures biocompatibility, rendering them suitable for food applications,^[^ ^22^ ^]^ while their high specific surface area and abundant active sites may facilitate their efficient coordination with natural preservatives such as α‐LA.^[^ ^23^ ^]^ However, traditional synthesis methods such as high‐temperature calcination (e.g., pyrolysis at 800 °C for fabricating psaFeN nanozymes)^[^ ^24^ ^]^ often damage the structure and functionality of natural preservatives, thereby reducing their bioactivity. Similarly, conventional wet‐chemical coordination methods involving multiple steps and strong reductants (e.g., NaBH_4_ for Pt‐ZnO nanocomposites)^[^ ^25^ ^]^ can be equally detrimental due to their harsh conditions. Moreover, these conventional coordination methods fail to fully integrate and optimize the synergistic effects of single‐atom nanozymes and preservatives, limiting their combined performance. Therefore, developing simple, mild and efficient coordination strategies to achieve controlled integration while preserving functional properties and enabling synergistic enhancement is a critical research focus. The electronic structure and catalytic activity of these materials can be precisely tuned by modulating their coordination environment to pave the way for high‐performance applications. Future advancements in green synthesis and interdisciplinary research are expected to expand the applications of these materials in energy, environmental, biomedicine, and food preservation, further driving innovations in these fields.

Recent progress in materials science and nanotechnology has led to rapid advancements in film packaging technology for food preservation. The focus in this field has shifted from traditional materials to high‐performance, intelligent, and sustainable solutions, particularly eco‐friendly bio‐based active packaging, to extend the shelf life of fruit.^[^ ^26^ ^]^ Natural bio‐based polymers such as polysaccharides and proteins are widely used in food packaging materials. Among them, chitosan (CS) and gelatin (Gel) stand out as particularly promising matrix components due to their complementary functional properties. CS provides certain antimicrobial activity and mechanical robustness, while Gel contributes excellent film‐forming capability, UV‐barrier capacity, and biocompatibility. However, when used individually, pure Gel films demonstrate limitations in mechanical strength and antimicrobial capacity, while single‐component CS films face challenges in processability and functional versatility.^[^ ^27^ ^]^ By combining CS and Gel into a composite matrix, this system may synergistically integrate their respective advantages while overcoming their individual limitations, thereby creating an enhanced material platform for advanced food packaging applications. Additionally, a noteworthy development in this field is the use of pH‐responsive materials, which can adapt their structures to external pH changes, enabling the intelligent release of active compounds. For example, researchers have developed pH‐responsive systems such as cinnamaldehyde‐based emulsion gels,^[^ ^28^ ^]^ chitosan‐oxidized fucoidan coatings,^[^ ^29^ ^]^ and ZIF‐8/TOCNF‐pectin carriers^[^ ^30^ ^]^ to extend the shelf life of fruits such as longan, citrus, litchi, raspberry, and mango. Smart coatings like α‐LA@Cu‐MOF^[^ ^31^ ^]^ exhibit dual pH‐ and humidity‐responsive release, leveraging the acidic microenvironment formed during fruit storage owing to metabolic activity and microbial growth. While these advances demonstrate significant progress, there remains a clear need for novel materials that not only possess intelligent release capabilities, but also integrate high functional activity to actively combat spoilage factors throughout the preservation period. The integration of multifunctional properties, including mechanical strength, barrier performance, antimicrobial and antioxidant capacities, and controlled release, into film packaging materials is a critical research priority. This approach would help maximize the preservation efficacy of such materials by precisely regulating the release of active compounds, thereby paving the way for the development of next‐generation food preservation technologies.

**Scheme 1:**
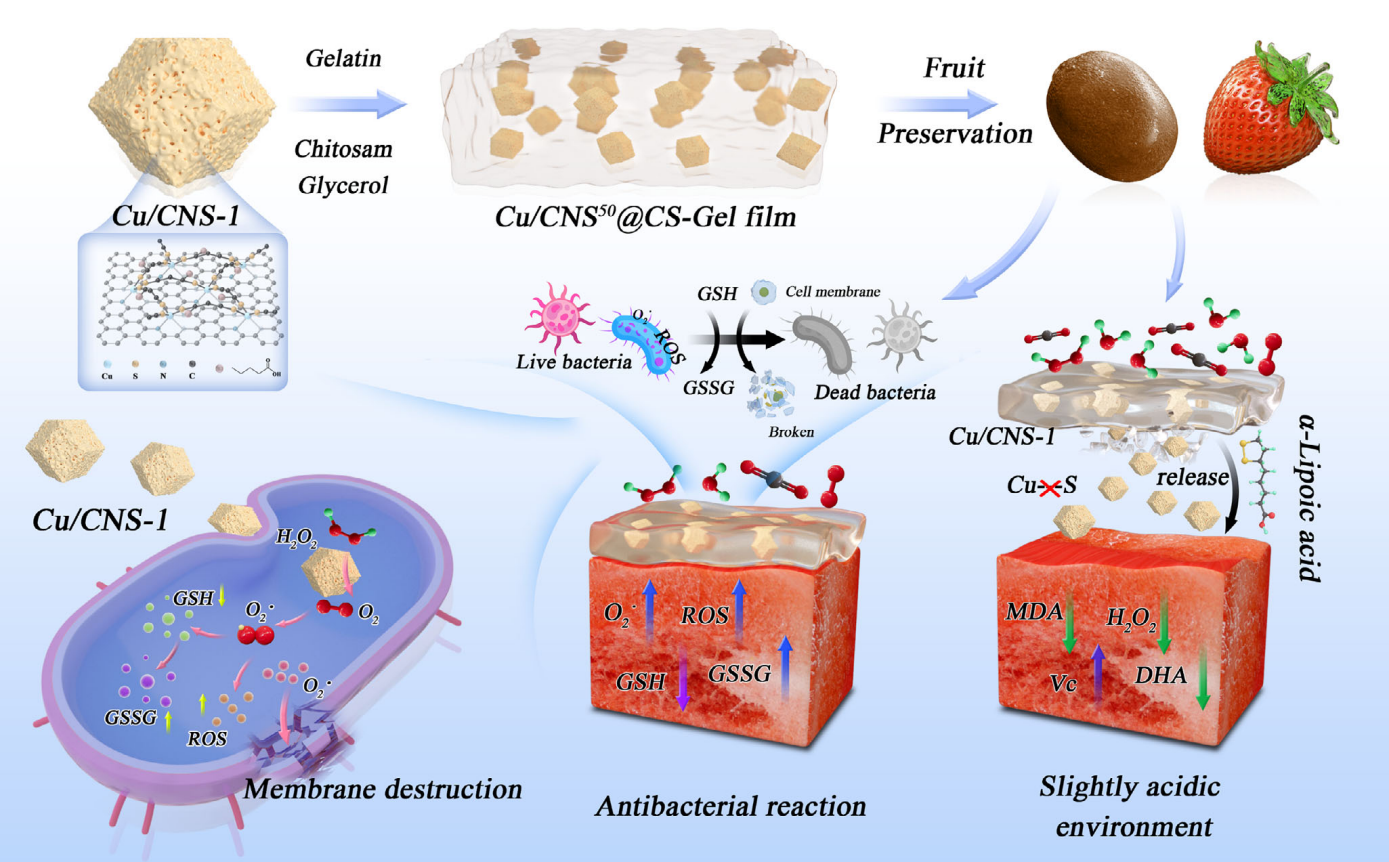
Schematic illustration of pH‐responsive release and synergistic antimicrobial mechanisms of Cu/CNS^50^@CS‐Gel film for fruit preservation.

This work establishes a pioneering photocatalysis‐driven self‐assembly strategy between α‐LA and Cu single‐atom nanozymes (Cu/CN) to obtain S‐coordinated Cu single‐atom nanozymes (Cu/CNS) with high dispersibility and multienzyme mimicry. The resulting Cu/CNS@CS‐Gel film represents a transformative advancement in active food packaging. It integrates pH‐responsive release, broad‐spectrum antimicrobial action (nearly 100% efficacy against *Escherichia coli*, *Staphylococcus aureus*, and *Botrytis cinerea*), and robust physicochemical stability, a combination previously unattained in sustainable preservation materials. By demonstrating dynamic catalytic enhancement (catalase (CAT)‐, oxidase (OXD)‐, and glutathione oxidase (GSH‐Ox)‐like activities) and universal compatibility with climacteric and nonclimacteric fruits, this study highlights the potential of single‐atom nanozymes in food science. Precise atom‐level coordination can not only overcome the traditional limitations of metal aggregation and reactivity loss, but also provide a generalizable design principle for next‐generation biohybrid materials. In addition to immediate applications in shelf life extension, this study opens up new avenues for smart enzyme‐mimetic packaging systems. Such advanced systems can replace synthetic preservatives and reduce food waste, which are critical challenges for sustainable development. Fundamentally, these findings establish crucial connections across nanocatalysis, food chemistry, and materials science. This integrated perspective provides a clear roadmap for developing high‐performance and sustainable alternatives to current agro‐food technologies (**Scheme** **1**).

## 2 Results and Discussion

### 2.1 Synthesis and Characterization of Cu/CNS‐1

**Figure 1:**
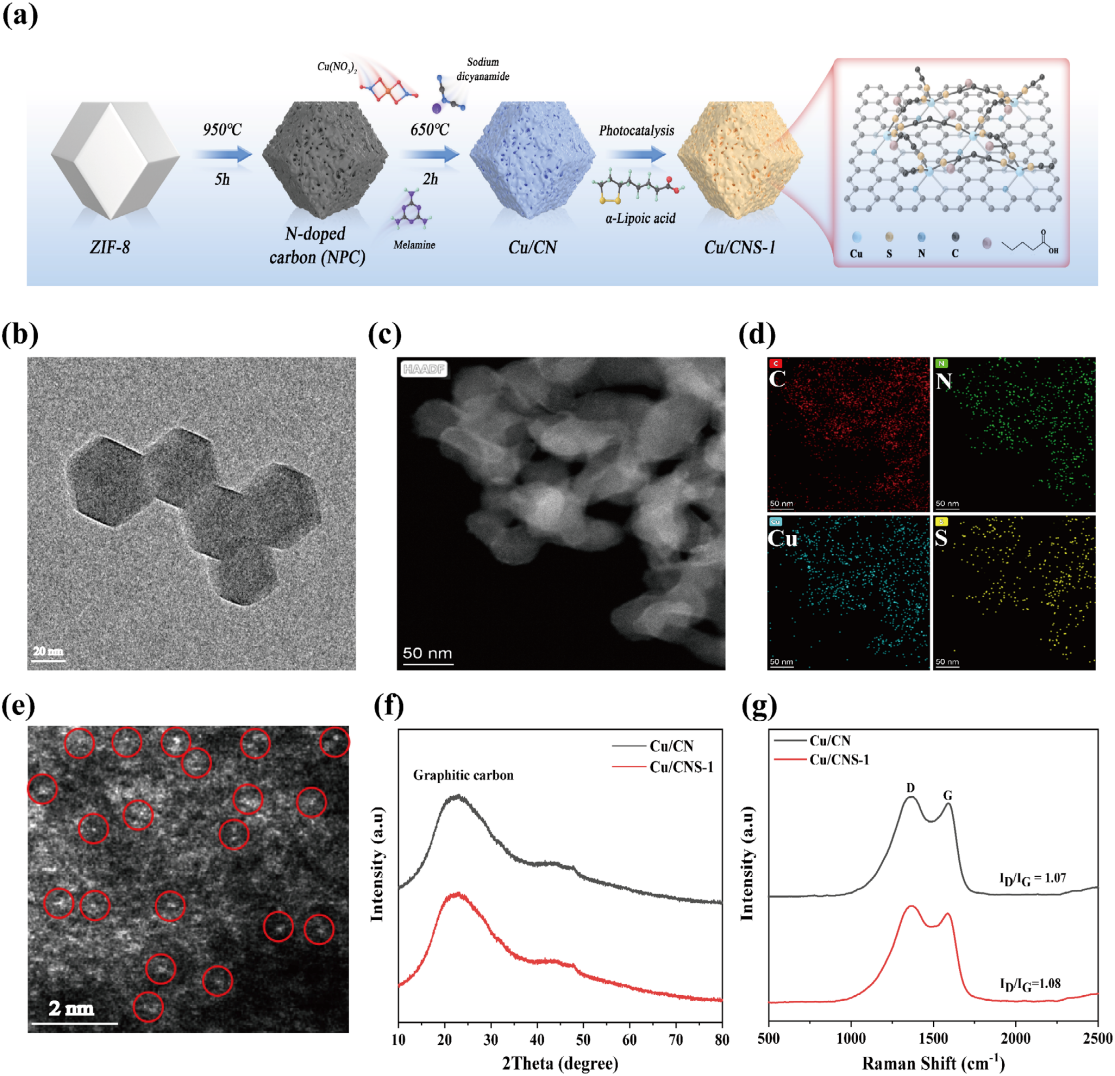
The schematic illustration synthesis of Cu/CNS‐1 by the photocatalytic‐driven coordination strategy a). TEM image b), HAADF‐STEM image c) and corresponding EDS mapping images d), AC‐HAADF‐STEM image e), XRD spectrum f), and Raman spectrum g) of Cu/CNS‐1.

The synthesis of Cu/CNS‐1 was achieved using a three‐step strategy involving high‐temperature calcination, chemical adsorption, and photocatalytic coordination (**Figure** **1**a). Initially, N‐doped porous carbon (NPC) with a high specific surface area and an abundant pore structure was prepared by calcining ZIF‐8 precursors in a tube furnace. Subsequently, Cu^2+^ and organic ligands were introduced into the NPC framework via chemical adsorption, followed by high‐temperature calcination to precisely anchor single‐atom Cu to the N‐doped sites. Finally, the disulfide bonds in α‐LA were cleaved by light energy under photocatalytic conditions to generate thiol groups, thereby enabling controlled coordination with the Cu single‐atom active sites. This method successfully produced structurally clear Cu/CNS‐1. The proposed strategy not only achieved the efficient loading of α‐LA but also precisely modulated the surface chemistry of the material through photocatalytically driven coordination, thus providing new insights into the rational design of multifunctional catalytic materials.

Transmission electron microscopy (TEM) revealed that Cu/CNS‐1 maintained a regular dodecahedral morphology with a uniform size distribution (Figure 1b). High‐angle annular dark‐field scanning TEM (HAADF‐STEM) and the corresponding EDS mapping confirmed the homogeneous distribution of C, N, O, S, and Cu in Cu/CNS‐1 (Figure 1c,d). The Aberration‐corrected HAADF‐STEM (AC‐HAADF‐STEM) images also verified the absence of Cu clusters or nanoparticles in Cu/CNS‐1, with isolated bright spots (marked by red circles) clearly indicating the presence of Cu in a single‐atom form^[^ ^32^, ^33^ ^]^ (Figure 1e). The X‐ray diffraction (XRD) patterns showed no characteristic peaks of Cu or its oxides, except for a broad peak near 25° corresponding to graphitic carbon; this result confirms the atom‐level dispersion of Cu within the NPC matrix, which is consistent with the AC‐HAADF‐STEM results^[^ ^34^, ^35^ ^]^ (Figure 1f). Raman spectroscopy revealed characteristic peaks at 1370.1 cm^−1^ (D‐band, carbon defects) and 1587.3 cm^−1^ (G‐band, sp^2^ hybridized carbon), with both bands shifting toward lower wavenumbers compared with those of Cu/CN^[^ ^36^, ^37^ ^]^ (Figure 1g). The *I*_D_/*I*_G_ ratios of Cu/CN and Cu/CNS‐1 were 1.07 and 1.08, respectively, indicating that the coordination process did not significantly alter the defect structure of the material^[^ ^38^ ^]^ (Table S1, Supporting Information). Inductively coupled plasma optical emission spectroscopy (ICP‐OES) analysis revealed that the mass percentages of Cu and S in Cu/CNS‐1 were 4.27% and 6.92%, respectively, demonstrating the efficiency of the proposed synthetic strategy (Table S2, Supporting Information). Fourier‐transform infrared (FTIR) spectroscopy and zeta potential analysis further confirmed the successful coordination of α‐LA to Cu sites via disulfide bond cleavage (Figures S1 and S2, Supporting Information). Brunauer–Emmett–Teller (BET) analysis indicated that Cu/CNS‐1 retained microporous and mesoporous structures with a high specific surface area (Figures S3–S5, Supporting Information). These results demonstrate that the photocatalytically driven coordination strategy not only enables the efficient integration of α‐LA with Cu single‐atoms but also prevents the formation of clusters or nanoparticles, thereby preserving the structural integrity and regularity of the material.

**Figure 2:**
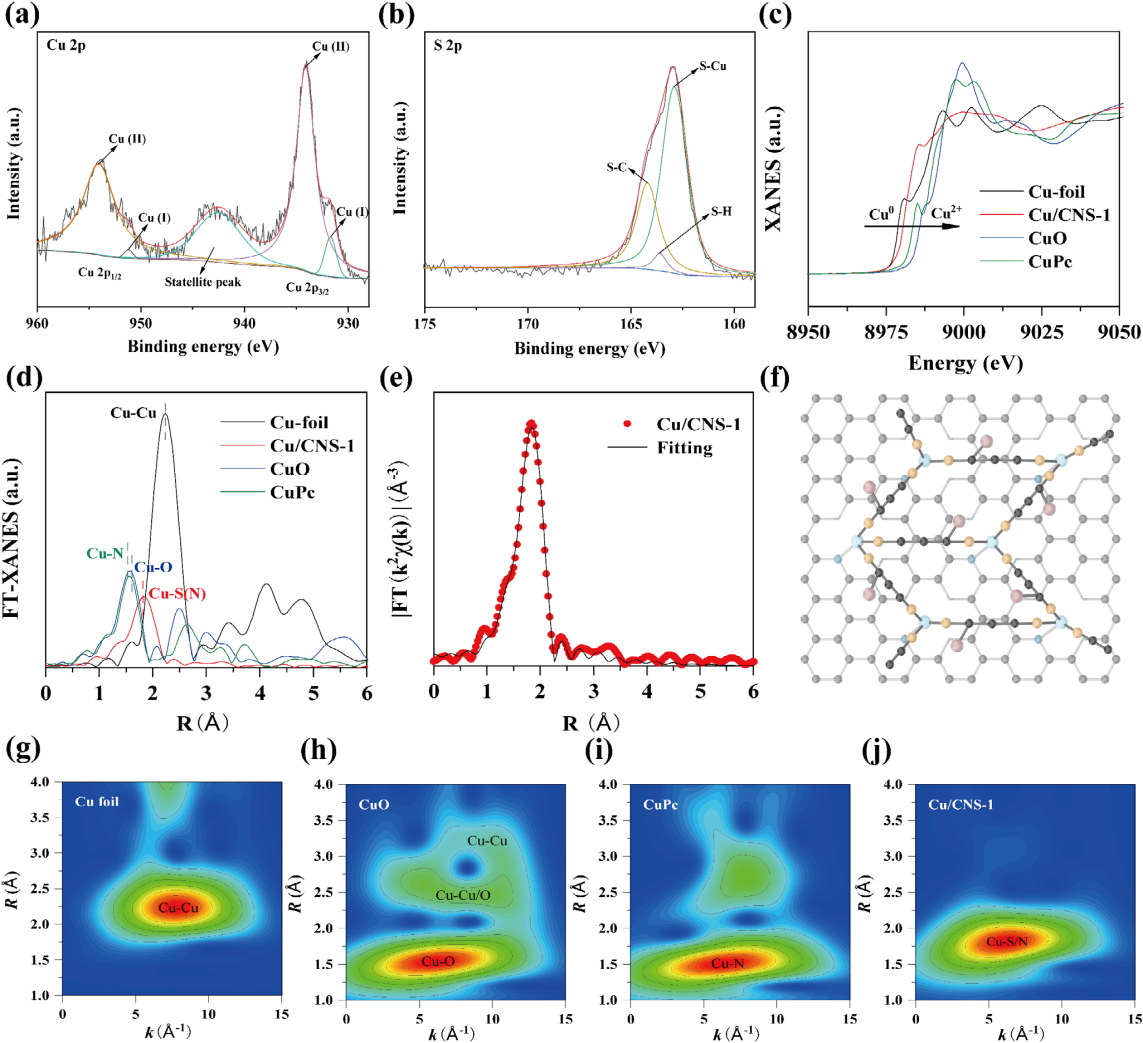
The XPS spectrum of Cu orbitals a) and S orbitals b) in Cu/CNS‐1, XANES spectrum of Cu/CNS‐1 and reference samples c). Fourier‐transform of the k^3^‐weighted Cu K‐edge EXAFS oscillation spectra of Cu/CNS‐1 and reference samples d). EXAFS fitting result of Cu/CNS‐1 in R space e). Schematic model of atomic level structure for Cu/CNS‐1 f). WT‐EXAFS plots of Cu/CNS‐1 and reference samples g–j).

The composition and valence states of Cu/CNS‐1 were thoroughly analyzed by X‐ray photoelectron spectroscopy (XPS). Compared with that of Cu/CN, the wide‐scan XPS profile of Cu/CNS‐1 exhibited a new peak at 162.98 eV, which was attributed to the S 2p orbital^[^ ^39^ ^]^ (Figure S6, Supporting Information). The high‐resolution Cu 2p spectrum revealed that the Cu 2p_3/2_ peak in Cu/CNS‐1 could be deconvoluted into a dominant peak at 934.1 eV and a minor peak at 931.7 eV, whereas the Cu 2p_1/2_ peak was fitted with a dominant peak at 954.1 eV and a minor peak at 951.2 eV.^[^ ^40^ ^]^ A satellite peak was observed at 942.6 eV^[^ ^39^ ^]^ (**Figure** **2**a). The high‐resolution S 2p spectrum revealed peaks at 162.9 and 163.6 eV, corresponding to S─Cu and S─H coordination bonds, respectively^[^ ^41^ ^]^ (Figure 2b). The high‐resolution N 1s spectrum indicated three primary N species in Cu/CNS‐1: Cu─N (397.8 eV), pyridinic N (399.0 eV), and graphitic N (400.6 eV)^[^ ^42^ ^]^ (Figure S8b, Supporting Information). These results collectively confirm the successful coordination of thiol groups (–SH), which were generated from the cleavage of disulfide bonds in α‐LA, with the Cu atoms in Cu/CN.

X‐ray absorption fine structure spectroscopy (XAFS), including X‐ray absorption near‐edge structure (XANES) and extended X‐ray absorption fine structure (EXAFS), was used to investigate the electronic structure and coordination environment of Cu and further elucidate the fine structure of Cu/CNS‐1. The Cu K‐edge XANES spectrum indicated that the absorption edge of Cu/CNS‐1 lay between those of Cu foil and CuPc, indicating that the oxidation state of Cu is between 0 and +2 (Figure 2c). The Fourier‐transform EXAFS spectrum of Cu/CNS‐1 exhibited a main peak that was attributed to Cu─N and Cu─S coordination (Figure 2d). Fitting of the synchrotron data revealed that the first‐shell coordination numbers for Cu─N and Cu─S were 0.7 (1.98) and 3.3 (2.27 Å), respectively, suggesting that the structural unit of Cu/CNS‐1 consists of one Cu atom, one N atom, and three S atoms (Figure 2e,f; Table S3, Supporting Information). Wavelet transform (WT) analysis confirmed the atom‐level dispersion of Cu species in Cu/CNS‐1, with a single intensity maximum, in stark contrast to the WT spectra of Cu foil, CuO, and CuPc (Figure 2g–j). In summary, the photocatalytically driven strategy introduced in this study could successfully synthesize Cu/CNS‐1 with a Cu/N_1_S_3_ structural unit, providing critical insights into the precise design of atomically dispersed catalysts.

### 2.2 Multienzyme Mimetic Activities of Cu/CNS‐1

**Figure 3:**
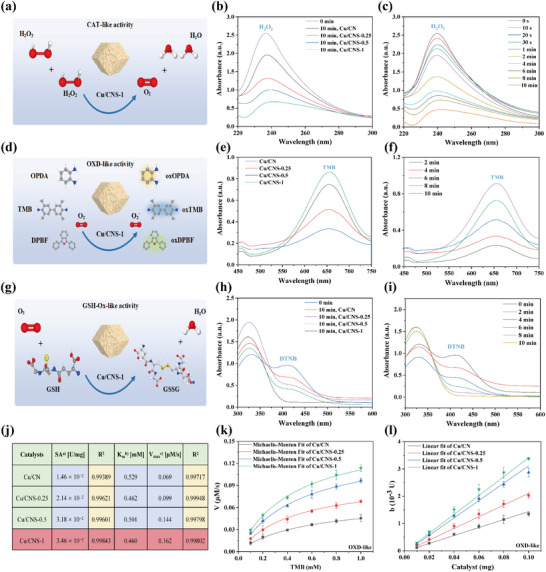
The schematic illustration of CAT‐like activity of Cu/CNS‐1 a). UV‐vis absorption spectra of H_2_O_2_ in the presence of Cu/CN, Cu/CNS‐0.25, Cu/CNS‐0.5, and Cu/CNS‐1 b). Time‐dependent absorption values changes of H_2_O_2_ in the presence of Cu/CNS‐1 c). The schematic illustration of OXD‐like activity of Cu/CNS‐1 d). UV‐vis absorption spectra of TMB in the presence of Cu/CN, Cu/CNS‐0.25, Cu/CNS‐0.5, and Cu/CNS‐1 e). Time‐dependent absorption values changes of TMB in the presence of Cu/CNS‐1 f). The schematic illustration of GSH‐Ox‐like activity of Cu/CNS‐1 g). UV‐vis absorption spectra of DTNB in the presence of Cu/CN, Cu/CNS‐0.25, Cu/CNS‐0.5, and Cu/CNS‐1 h). Time‐dependent absorption values changes of DTNB in the presence of Cu/CNS‐1 i). The comparison of the OXD‐kinetic constants of Cu/CN, Cu/CNS‐0.25, Cu/CNS‐0.5 and Cu/CNS‐1 j). Steady‐state kinetics of Cu/CN, Cu/CNS‐0.25, Cu/CNS‐0.5, and Cu/CNS‐1 for substrate TMB k). Specific activities of Cu/CN, Cu/CNS‐0.25, Cu/CNS‐0.5, and Cu/CNS‐1 for substrate TMB (l).

The multienzyme mimetic activities of Cu/CNS‐1, including its CAT‐, OXD‐, and GSH‐Ox‐like activities, were systematically evaluated. First, the CAT‐like activity of Cu/CNS‐1 was assessed by monitoring the characteristic absorption peak of hydrogen peroxide (H_2_O_2_) at 240 nm. Compared with Cu/CN, Cu/CNS‐0.25, and Cu/CNS‐0.5, Cu/CNS‐1 exhibited significantly higher efficiency in catalyzing the decomposition of H_2_O_2_ into O_2_ and H_2_O. After 10 min of reaction, the characteristic peak of H_2_O_2_ nearly disappeared, confirming the superior CAT‐like activity of Cu/CNS (**Figure** **3**a–c). This property provides the necessary conditions for triggering subsequent OXD‐like activity, which was subsequently evaluated using 3, 3′, 5, 5′‐tetramethylbenzidine (TMB), o‐phenylenediamine (OPDA), and 1, 3‐diphenylisobenzofuran (DPBF) as substrates. Cu/CNS‐1 significantly accelerated the oxidation of TMB, turning the solution from colorless to deep blue, with a 157.58% increase in the intensity of the characteristic absorption peak at 652 nm compared with Cu/CN. This result indicates that Cu/CNS‐1 efficiently catalyzes the oxidation of TMB to oxidized TMB (oxTMB) (Figure 3d–f). Key enzymatic parameters, including *K*_m_ and *V*_max_, were calculated using the classical Michaelis–Menten equation and TMB as the substrate (Figure 3j). Compared with Cu/CN, Cu/CNS‐0.25, and Cu/CNS‐0.5, Cu/CNS‐1 exhibited a significantly lower *K*_m_ (0.46 mM) and higher *V*_max_ (0.162 µM/s). The *V*_max_ of Cu/CNS‐1 was 134.78% higher than that of Cu/CN, demonstrating its superior substrate affinity and reaction kinetics (Figure 3k). The SAs of the four nanozymes were calculated to further evaluate their catalytic performance. The specific activity (SA) values of Cu/CN and Cu/CNS‐1 were 1.46 × 10^−2^ and 3.46 × 10^−2^ U mg^−1^, respectively, representing a 136.99% increase for Cu/CNS‐1 compared with Cu/CN (Figure 3l). When the activity was normalized to a single catalytic site, the SAs were calculated to be 2.84 × 10^−20^ U per atom Cu (2.85× 10^−4^ s^−1^) and 8.56 × 10^−20^ U per atom Cu (8.59× 10^−4^ s^−1^) for Cu/CN and Cu/CNS‐1, which corresponded to a remarkable enhancement of 201.41% at the level of a single atom. Similarly, Cu/CNS‐1 exhibited significantly enhanced oxidation capabilities for OPDA and DPBF, as evidenced by notable changes in the characteristic peaks observed at 450 and 420 nm, further confirming its outstanding OXD‐like activity (Figures S12 and S13, Supporting Information).

To further validate the catalytic pathway under the endogenous H_2_O_2_ (≈3 mm) concentration calculated based on fruit tissue water content,^[^ ^43^ ^]^ we performed additional enzyme activity assays. As shown in Figure S14 (Supporting Information), under ambient conditions, the Cu/CNS‐1 + TMB + H_2_O_2_ (3 mm) system exhibited the strongest chromogenic response, which was only slightly weakened upon the introduction of a hydroxyl radical (•OH) scavenger. It indicates that the CAT‐OXD cascade likely predominates the catalytic process at this H_2_O_2_ level, with POD‐like activity playing a relatively minor role. Figure S15 (Supporting Information) demonstrates that in an O_2_‐free N_2_ atmosphere, the Cu/CNS‐1 + TMB system showed almost no color change, while the Cu/CNS‐1 + TMB + H_2_O_2_ (3 mm) system still produced a significant colorimetric signal, which was only slightly reduced upon the addition of a •OH scavenger. These results provide strong support for the conclusion that the CAT‐like decomposition of H_2_O_2_ generates localized O_2_, which in turn drives the OXD‐like activity, thereby establishing a self‐sustaining catalytic cycle, and further confirm the relatively weak contribution of POD‐like activity in this system. In summary, Cu/CNS‐1 may operate through a well‐coordinated CAT‐OXD catalytic cascade that can be effectively initiated and sustained at endogenous H_2_O_2_ concentrations. And the unique electronic structure of the Cu‐N_1_S_3_ active center, characterized by its electron‐rich Cu‐S_3_ core resulting from sulfur coordination, may intrinsically favor the two‐electron transfer pathway required for CAT‐like activity while impeding the stabilization of •OH intermediates essential for POD‐like reactions, thereby establishing the preferential selection of CAT‐like over POD‐like activity.^[^ ^44^ ^]^ Furthermore, this structural specificity potentially contributes to the efficient decomposition of fruit‐derived H_2_O_2_ into O_2_ through CAT‐like activity, which serves dual roles of eliminating a potential pro‐oxidant while providing the essential substrate for subsequent OXD‐like activity.^[^ ^45^ ^]^ This OXD‐like activity then converts the locally generated O_2_ into antibacterial superoxide anions (•O_2_−) capable of disrupting cellular membranes. Through this self‐sustaining cycle that capitalizes on intrinsic fruit metabolites, Cu/CNS‐1 maintains continuous production of antibacterial •O_2_−, demonstrating significant potential for antimicrobial applications and fruit preservation.

Glutathione (GSH), a crucial intracellular antioxidant in microorganisms, neutralizes ROS, thereby reducing ROS‐based antimicrobial efficiency.^[^ ^46^ ^]^ GSH depletion can significantly enhance ROS‐mediated antimicrobial effects.^[^ ^47^ ^]^ The GSH‐Ox‐like activity of Cu/CNS‐1 was evaluated using 5, 5′‐dithiobis‐(2‐nitrobenzoic acid) (DTNB) as a probe. Compared with Cu/CN, Cu/CNS‐0.25, and Cu/CNS‐0.5, Cu/CNS‐1 more efficiently oxidized GSH to GSSG and H_2_O. After 10 min of reaction, the characteristic absorption peak at 420 nm nearly disappeared, indicating the nearly complete depletion of GSH by Cu/CNS‐1 (Figure 3g–i). This result confirms the pronounced GSH‐Ox‐like activity of Cu/CNS‐1, which enables efficient GSH depletion and promotes ROS accumulation, thereby enhancing the antimicrobial efficiency of the material.

Overall, the enhancement of the enzyme‐like activities could be attributed to the photocatalytic cleavage of disulfide bonds in α‐LA, which generated thiol groups enabling controlled coordination with Cu single‐atom sites. The precise photocatalytic‐activation strategy fundamentally transformed the nanozyme surface into a highly efficient platform for ROS generation. The integrated α‐LA moiety enhanced the intrinsic nanozyme activity through a synergistic electronic effect where it functioned simultaneously as an electron‐withdrawing ligand that modulated the electronic density of the Cu active center to establish more favorable redox cycling, and as an efficient electron‐transfer mediator that promoted catalytic turnover. This electronic synergy, combined with the inherent antioxidant and antibacterial properties of α‐LA, collectively amplified the ROS flux, thereby directly accounting for the superior antibacterial performance and fruit preservation efficacy demonstrated in our subsequent study. Furthermore, the enzyme‐like activities (CAT, OXD, and GSH‐Ox) of the four nanozymes showed the order Cu/CNS‐1 > Cu/CNS‐0.5 > Cu/CNS‐0.25 > Cu/CN. As the reaction time increased, Cu/CNS‐1 efficiently catalyzed the conversion of H_2_O_2_, TMB, and GSH, thus confirming its remarkable multienzyme mimetic activities. This multienzyme synergistic mechanism provides a solid theoretical foundation for the applications of Cu/CNS‐1 in antimicrobial fields and opens up new research directions for its potential use in food preservation.

### 2.3 In Vitro Antimicrobial Capacity and Mechanism

**Figure 4:**
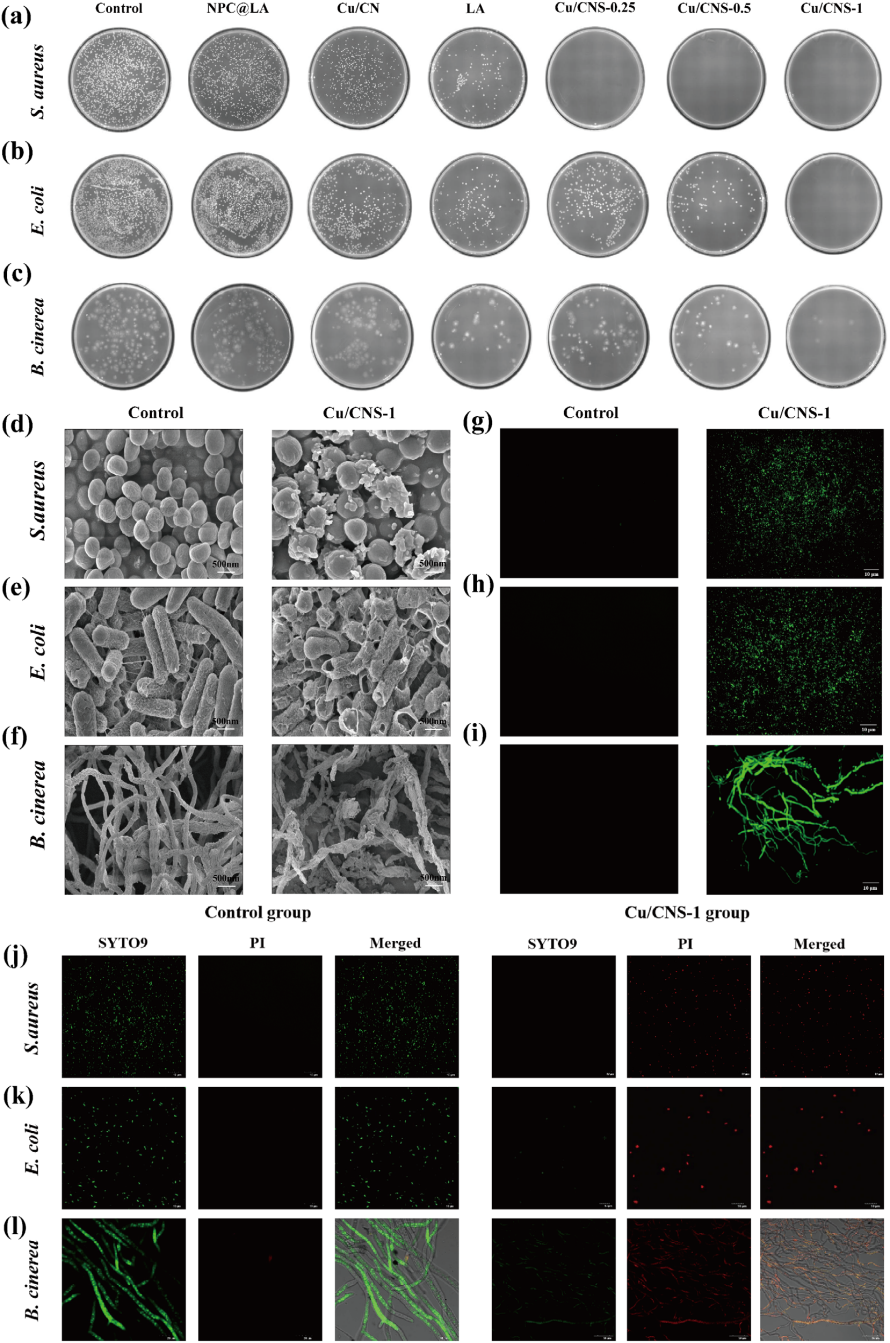
The antimicrobial capacities against *S. aureus*, *E. coli*, and *B. cinerea* of NPC@LA, Cu/CN, LA, Cu/CNS‐0.25, Cu/CNS‐0.5, and Cu/CNS‐1 a–c). The microbial morphology d–f), ROS levels g–i), live/dead staining j–l) of *S. aureus*, *E. coli*, and *B. cinerea* after Cu/CNS‐1 treatment.

The antimicrobial performance of Cu/CNS‐1 was evaluated based on its inherent multienzyme mimetic activity. The in vitro antimicrobial efficacy of Cu/CNS‐1 was comprehensively investigated using colony counting assays, cell viability tests, and DNA leakage analysis with *Escherichia coli* (*E. coli*), *Staphylococcus aureus* (*S. aureus*), and *Botrytis cinerea* (*B. cinerea*) as models (**Figure** **4**a–c; Figure S16, Supporting Information). NPC@LA exhibited negligible antimicrobial capacity, whereas Cu/CN demonstrated moderate antibacterial effects against *E. coli* and *S. aureus* (50.6% and 58.2%, respectively) as well as weak antifungal activity against *B. cinerea* (21.9%). α‐LA showed uniform antimicrobial capacity against all three microorganisms (75.6%, 76.7%, and 70.2%). Compared with NPC@LA, Cu/CN, and α‐LA, Cu/CNS exhibited broader spectrum and more efficient antimicrobial performance, with efficacy increasing progressively with its concentration. Notably, Cu/CNS‐1 demonstrated potent antimicrobial effects against all three microorganisms, achieving inhibition rates of 99.9%, 100%, and 97.7% for *E. coli*, *S. aureus*, and *B. cinerea*, respectively. Further analysis revealed that Cu/CNS‐1 treatment significantly reduced microbial cell viability and increased DNA leakage levels, confirming its broad‐spectrum antimicrobial efficacy. The enhanced performance of this treatment is likely attributable to the photocatalytically driven coordination strategy, which not only significantly improved the enzymatic activity of Cu/CNS‐1 but also fully leveraged the antimicrobial properties of α‐LA, resulting in a synergistic antimicrobial effect. Collectively, the results indicate that the antimicrobial performance of Cu/CNS is independent of the NPC substrate, confirming that α‐LA acts on the Cu active sites to achieve superior synergistic antimicrobial effects. Additionally, among the treatments investigated, Cu/CNS‐1 exhibited the strongest antimicrobial activity against both bacteria and fungi, far surpassing those of the other treatment groups. This discovery not only provides new insights for developing high‐performance antimicrobial materials but also lays a critical foundation for addressing microbial contamination in food preservation. Among the three Cu/CNS variants prepared with different α‐LA ratios, Cu/CNS‐1 demonstrated the most significant in vitro antimicrobial capacity and was selected for further investigation.

Systematic studies were conducted from four perspectives, namely, microbial morphology, ROS levels, live/dead staining, and molecular‐level evidence, to comprehensively elucidate the antimicrobial mechanisms of Cu/CNS‐1. SEM observations revealed that Cu/CNS‐1 significantly disrupted the cellular structures of both bacteria and fungi (Figure 4d–f). In *E. coli* and *S. aureus*, Cu/CNS‐1 treatment caused noticeable surface collapse and rupture, severe deformation of the cytoskeleton, and compromised cell membrane integrity. Similarly, significant morphological changes, such as shrinkage and structural breakage, were observed in the hyphae of *B. cinerea*. Further detection using DCFH‐DA as the probe showed that Cu/CNS‐1 treatment increased ROS levels in bacteria and fungi, as indicated by the enhanced intensity of the green fluorescence signals (Figure 4g–i). A sharp increase in oxidative stress is a key factor that contributes to microbial death. The apoptosis levels of the microorganisms were assessed using PI/SYTO9 dual staining to validate these findings (Figure 4j–l). A significant increase in red fluorescence (dead cells) and marked decrease in green fluorescence (live cells) in the Cu/CNS‐1 treatment group, consistent with the ROS detection results, were observed. Furthermore, to substantiate the oxidative damage at the molecular level, lipid peroxidation and the activity of key antioxidant enzymes were evaluated. As shown in Figure S17 (Supporting Information), Cu/CNS‐1 treatment significantly elevated the level of malondialdehyde (MDA) in bacteria and fungi, indicating severe peroxidative damage to membrane lipids. This was further corroborated by the assessment of critical antioxidant enzyme activities (Figure S18, Supporting Information). The activities of superoxide dismutase (SOD), catalase (CAT), peroxidase (POD), and glutathione peroxidase (GSH‐Px) were markedly suppressed in the Cu/CNS‐1 treated groups compared to the controls. The concerted inhibition of these essential defense enzymes effectively disabled the microbial antioxidant system, thereby amplifying intracellular oxidative stress and leading to irreversible metabolic dysfunction and cell death.

Based on these experimental findings, the overall antimicrobial mechanism of Cu/CNS‐1 could be summarized as follows: (1) generation of •O_2_− through OXD‐like activity and (2) depletion of intracellular GSH via GSH‐Ox‐like activity. These combined effects disrupt the redox balance, leading to cell membrane rupture and massive ROS accumulation, ultimately causing microbial death. The multifaceted antimicrobial mechanism of Cu/CNS‐1 is systematically elucidated in this study for the first time, providing critical theoretical support for its application in food preservation.

### 2.4 Characterization of Film Properties

**Figure 5:**
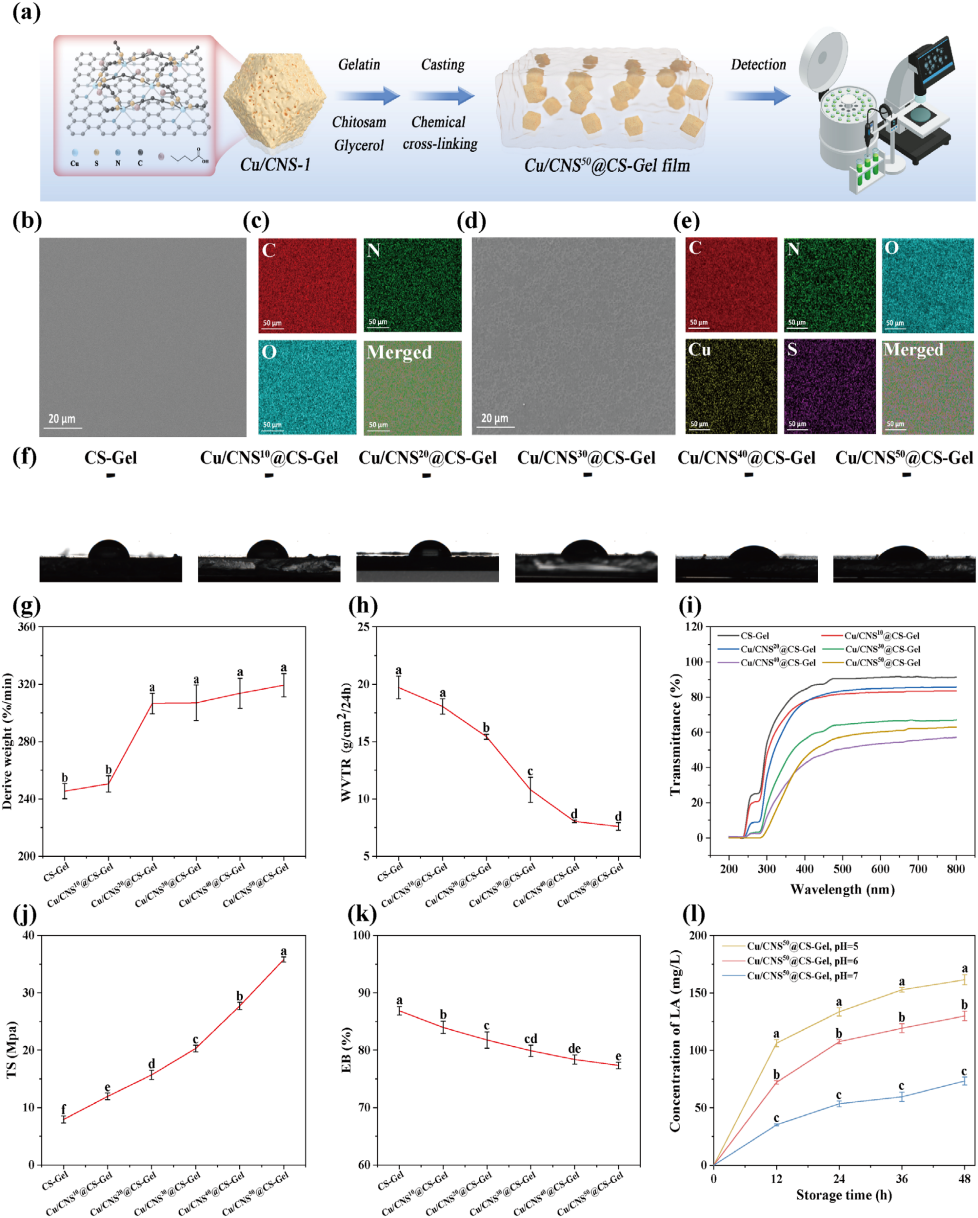
The schematic diagram of film preparation a), SEM and element mapping diagram of CS‐Gel and Cu/CNS^50^@CS‐Gel film b–e), WCA f), derive weight g), WVYR h), UV‐barrier property i), TS j), EB k) of CS‐Gel film, Cu/CNS^10^@CS‐Gel film, Cu/CNS^20^@CS‐Gel film, Cu/CNS^30^@CS‐Gel film, Cu/CNS^40^@CS‐Gel film and Cu/CNS^50^@CS‐Gel film. Controlled release behavior of α‐LA (l) in Cu/CNS^50^@CS‐Gel film under different pH conditions (pH 5, 6, and 7). Means followed by different letters are significantly different at *p* < 0.05.

Cu/CNS@CS‐Gel composite films were prepared using a casting method combined with chemical crosslinking (**Figure** **5**a). SEM analysis revealed dense, nonporous structures, indicating excellent compatibility between Gel, CS, and Cu/CNS‐1. The Cu/CNS@CS‐Gel film exhibited a unique textured surface, with a uniform dispersion of 50 mg of Cu/CNS‐1 optimizing its material properties (Figure 5b–e). The enhanced hydrophilicity of the film, as evidenced by its low water contact angle (WCA) value, was attributed to the polar groups (e.g., carboxyl and thiol) on Cu/CNS‐1, the cleavage of nonpolar disulfide bonds during α‐LA coordination, and the occurrence of synergistic interactions between Cu/CNS‐1 and the matrix^[^ ^48^ ^]^ (Figure 5f). FTIR spectroscopy confirmed that Cu/CNS‐1 incorporation did not disrupt the chemical structure of the CS‐Gel matrix, with the characteristic peaks indicating N–H bending and C═O stretching vibrations remaining intact^[^ ^49^ ^]^ (Figure S22, Supporting Information). Thermal stability, which was evaluated using thermogravimetric analysis, showed significant improvements in the degradation temperatures of Cu/CNS‐1. The degradation temperature of Cu/CNS^50^@CS‐Gel reached 319.3 and 73.9 °C higher than that of the control, owing to the occurrence of strong interfacial interactions (e.g., amide and hydrogen bonds) between Cu/CNS‐1 and the CS‐Gel matrix, a denser network structure restricting molecular chain motion, and the potential catalytic effects of Cu/CNS‐1 forming a carbonized layer^[^ ^50^, ^51^, ^52^ ^]^ (Figure 5g; Figures S25 and S26, Supporting Information). The barrier properties of films, which are crucial for food packaging, were systematically assessed. Cu/CNS^50^@CS‐Gel achieved near‐zero UV transmittance and the lowest water vapor transmission rates (WVTR) among the films investigated (Figure 5h,i). This improvement in UV barrier properties stems from the aromatic ring structure of α‐LA, which enhances UV absorption, Cu/CNS‐1 forming a UV scattering network, and synergistic effects.^[^ ^53^ ^]^ The low WVTR of the film was attributed to Cu/CNS‐1 creating a tortuous path for water diffusion, enhancements in interfacial interactions, and increases in film density.^[^ ^54^, ^55^ ^]^ Mechanical tests revealed that Cu/CNS‐1 exhibited significantly enhanced tensile strength (TS, up to 35.81 MPa) while maintaining flexibility with an elongation at break (EB) of 77.34%, as supported by the enhanced network structure stability of the film (Figure 5j,k). The results of DPPH and ABTS assays, which were conducted to reveal the antioxidant performance of the films, showed that Cu/CNS^50^@CS‐Gel achieved scavenging rates of 66.3% and 59.8%, respectively (Figures S27 and S28, Supporting Information). This enhancement was attributed to the Cu active sites and polar groups in Cu/CNS‐1 facilitating electron transfer and radical quenching, synergistic interactions improving antioxidant site accessibility, and the nanocomposite structure providing a larger surface area for radical adsorption and scavenging.^[^ ^9^, ^56^ ^]^ In general, the Cu/CNS^50^@CS‐Gel film exhibited integrated overall performance, including uniform dispersion, optimized hydrophilicity, improved thermal and mechanical properties, superior barrier capabilities, and strong antioxidant capacity, rendering it an ideal candidate for food packaging applications.

Meanwhile, the Cu/CNS^50^@CS‐Gel film exhibited excellent pH‐responsive properties, enabling precise control over α‐LA release in response to environmental pH changes, which is a critical feature for postharvest fruit preservation.^[^ ^57^ ^]^ Release kinetics studies revealed significant pH‐dependent behavior: at pH 7.0, 73.33 mg L^−1^ α‐LA was released over 48 h; at pH 6.0 and 5.0, α‐LA release increased to 129.96 and 161.49 mg L^−1^, respectively, representing increases of 77.3% and 120.3% (Figure 5l). This behavior stems from the enhanced swelling of the CS‐Gel matrix under acidic conditions, cleavage of Cu─S bonds, and weakened hydrogen bonding, all of which facilitate α‐LA diffusion. Among them, the cleavage of Cu─S bonds is primarily attributed to protonation of sulfur atoms under acidic conditions, which weakens the coordination interaction and promotes bond dissociation. The optimal release range of the film aligns with the slightly acidic environment (pH 5.0–6.0) formed on postharvest fruit surfaces during respiration, enabling intelligent α‐LA release: increased respiration promotes release to inhibit microbial growth, while decreased respiration slows release for precise control. Overall, the pH‐responsive multifunctional film offers an efficient and green solution for fruit preservation by combining biocompatibility, biodegradability, and intelligent release capabilities. Future research will focus on optimizing the mechanical properties of the prepared film and scaling up its production for industrial applications.

Additionally, the migration of metal ions from the Cu/CNS^50^@CS‐Gel film was further evaluated. As illustrated in Figure S29 (Supporting Information), the cumulative release of copper from the film was monitored over a period of 8 days. The results indicated a rapid initial release of Cu ions during the first two days, after which the migration reached a near‐plateau phase. By day 2, the released amount approached a near‐maximum value of ≈1.05 mg kg^−1^, increasing only marginally to 1.20 mg kg^−1^ by the end of the 8‐day period. This controlled and limited leaching behavior suggested that the Cu/CNS^50^@CS‐Gel film exhibited excellent structural stability, effectively retaining copper species and minimizing continuous ion release. The observed migration profile, characterized by an initial burst followed by rapid stabilization, rendered the material particularly suitable for applications requiring minimal metal leaching, such as food packaging. Importantly, the maximum detected copper migration level remained well below the specific migration limit of 5 mg kg^−1^ stipulated by the European Commission regulation (EU No 10/2011) for food‐contact materials. Hence, this result confirms the material's compliance with stringent safety standards and underscores its potential for safe practical deployment.

### 2.5 Application of the Cu/CNS^50^@CS‐Gel Coating Film to Fruit Preservation

Safety evaluations of single‐atom materials are crucial for their application in food preservation, ensuring biocompatibility and environmental friendliness to avoid potential toxicity. The safety profile of Cu/CNS‐1 was systematically evaluated through hemocompatibility and cytotoxicity assays. As shown in Figure S30 (Supporting Information), Cu/CNS‐1 exhibited significantly lower hemolysis rates across a wide concentration range (25–800 µg mL^−1^) compared to Cu/CN, with rates similar to those of NPC, demonstrating excellent biocompatibility. This favorable safety was further confirmed by cytotoxicity tests (Figure S31, Supporting Information), where at the highest concentration (800 µg/mL), Cu/CNS‐1 treatment maintained 89.03% cell viability, which was significantly higher than the 86.24% observed for Cu/CN and nearly equivalent to the 90.30% viability of the NPC control, and these values remained within the acceptable level according to ISO 10993‐5‐2009.^[^ ^58^ ^]^ The consistently high biosafety demonstrated across these independent biological evaluations strongly suggested that the structural integration of sulfur in the Cu/CNS‐1 framework effectively mitigated the potential toxicity associated with copper species while preserving the material's functional integrity. Overall, these results indicate that the photocatalytically driven coordination strategy effectively mitigates the inherent toxicity of Cu/CN, rendering it fully compliant with safety requirements for food preservation applications. This study provides an important theoretical foundation for the development of high‐performance and low‐toxicity functional materials.

**Figure 6:**
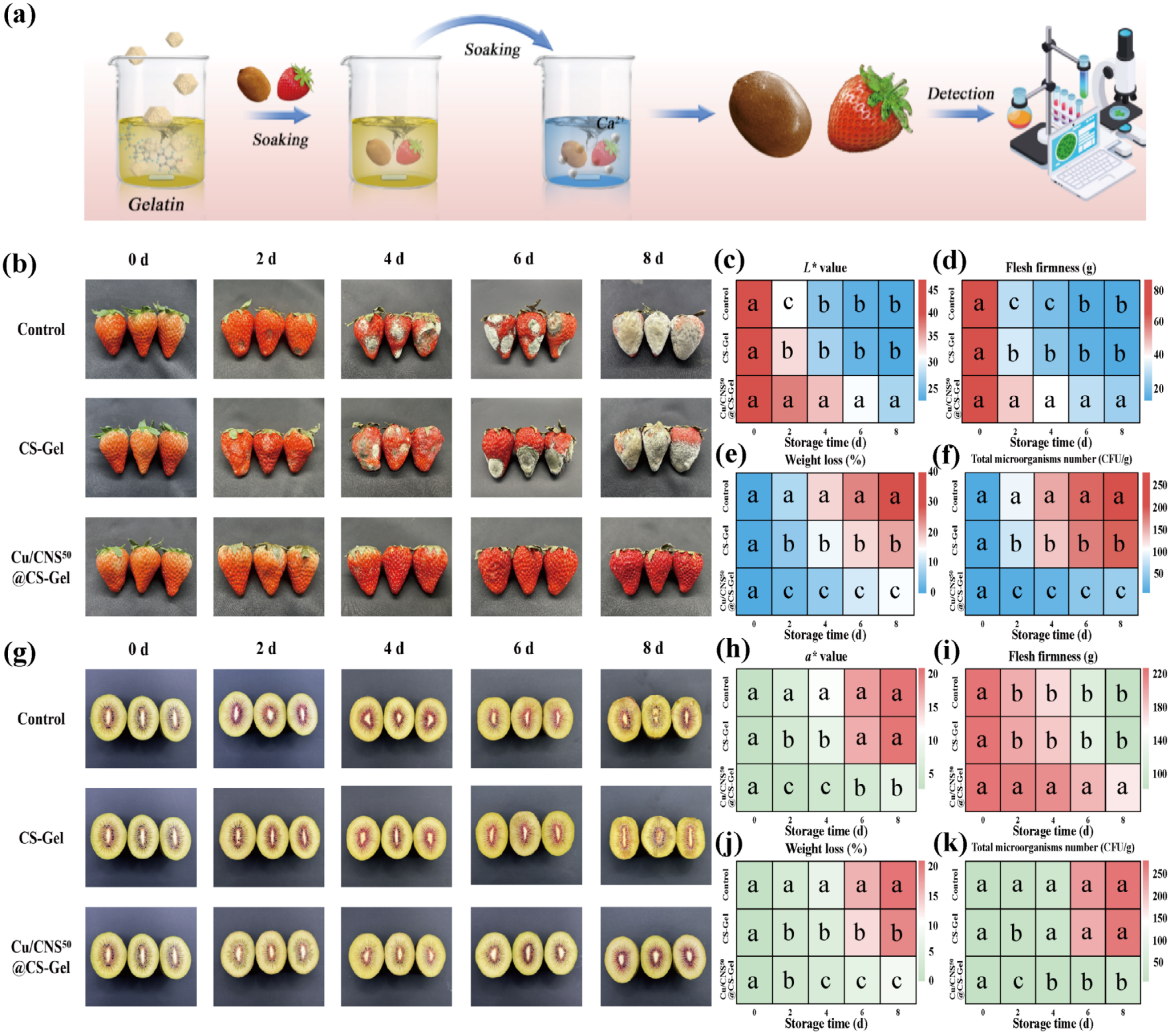
The schematic diagram of fruit preservation a), appearance b), L* value c), flesh firmness d), weight loss e), and the total microorganism number f) of strawberries. The appearance g), a* value h), flesh firmness i), weight loss j), and the total microorganism number k) of kiwifruits. Means followed by different letters are significantly different at *p* < 0.05.

The Cu/CNS^50^@CS‐Gel film demonstrated exceptional preservation efficacy for both nonclimacteric (strawberry) and climacteric (kiwifruit) fruits (**Figure** **6**a). The film maintained the excellent physical appearance of strawberries over 8 d, significantly delaying declines in L* value (mainly reflecting brightness changes), firmness, and nutritional quality while reducing weight loss and microbial growth (Figure 6b–f; Figures S33–S35, Supporting Information). These effects were attributed to the superior mechanical properties, barrier performance, and antimicrobial capacity of the film.^[^ ^59^ ^]^ Similarly, the film preserved the physical appearance of kiwifruits, inhibiting a* value changes (mainly reflecting degree of yellowing), slowing firmness decline, reducing weight loss, and maintaining antioxidant capacity and nutritional quality (Figure 6g–k; Figures S36,S38–S40, Supporting Information). The barrier properties of the film reduced respiration rates, while its good antioxidant capacity and generated free radicals degraded endogenous ethylene (Figure S41, Supporting Information), resulting in delayed ripening.^[^ ^60^, ^61^ ^]^ Furthermore, the migration of copper ions into the coated fruits was quantitatively evaluated. As illustrated in Figure S42 (Supporting Information), the internal copper ion content in strawberries (pulp) and kiwifruits (both peel and pulp) was monitored over an 8‐day storage period. The initial concentrations measured on day 0 were 0.57, 0.28, and 0.10 mg kg^−1^ (Table S6, Supporting Information), which increased only marginally to 1.52, 0.75, and 0.29 mg kg^−1^ by the end of day 8 (Table S7, Supporting Information). The observed stability in copper content was primarily governed by the robust chelation environment created by the nitrogen and sulfur co‐dopants within the carbon matrix. The formation of stable Cu‐N/S coordination bonds effectively immobilized the copper species at an atomic level, creating a high energy barrier for ion dissociation. This specific coordination chemistry rendered the copper centers kinetically inert against leaching, resulting in the very limited migration observed throughout the storage period. Summarily, these results demonstrate that the composite coating complies with the specific migration limits for these elements in food contact materials as stipulated by the European Commission regulation (EU No 10/2011), which sets a threshold of 5 mg kg^−1^ for copper. The observed copper ion migration, remaining well below this regulatory limit, underscores the material's suitability and safety for practical fruit preservation applications.

The preservation mechanism of the Cu/CNS^50^@CS‐Gel film involves multiple synergistic effects: (1) Cu/CNS‐1 acts as a CAT‐like nanozyme, generating O_2_ from H_2_O_2_; (2) as an OXD‐like nanozyme, it converts O_2_ into ·O_2_−, damaging microbial cell membranes and degrading ethylene; (3) as a GSH‐Ox‐like nanozyme, it depletes microbial GSH, promoting ROS accumulation; (4) the slightly acidic fruit surface promotes pH‐responsive α‐LA release, enhancing antioxidant and antimicrobial effects; and (5) the barrier properties of the film protect against UV damage and reduce respiration rates. Collectively, these mechanisms delay fruit ripening and senescence. Ultimately, the Cu/CNS^50^@CS‐gel film offers a multifunctional, efficient, and environmentally friendly solution for fruit preservation. To advance this technology, future research should focus on three critical dimensions: First, systematically evaluating the universal applicability of this coordination chemistry across diverse metal‐ligand systems and its long‐term catalytic stability under real‐world storage conditions. Second, engineering advanced functionalities such as targeted ethylene adsorption/degradation or reactive oxygen species scavenging to address species‐specific spoilage pathways. Most importantly, integrating multi‐omics approaches (single‐cell transcriptomics, phosphoproteomics, and volatile metabolomics) could unravel conserved molecular networks governing fruit responses to the film, particularly in key regulatory pathways related to ripening, stress tolerance, and nutritional quality retention. Such mechanistic insights would not only refine material design principles but also establish predictive models for tailoring preservation strategies across climacteric and non‐climacteric species—a crucial step toward sustainable postharvest management.

## 3 Conclusion

In summary, this study successfully developed a multifunctional antimicrobial platform, Cu/CNS‐1, via a photocatalysis‐driven self‐assembly coordination strategy. Cu/CNS‐1 exhibited integrated enzyme‐like activities (CAT, OXD, and GSH‐Ox), achieving nearly 100% broad‐spectrum antimicrobial activity against bacteria and fungi. A composite film with a dense network structure was constructed by integrating Cu/CNS‐1 into a CS‐Gel matrix. The Cu/CNS^50^@CS‐Gel film exhibited superior mechanical performance, barrier properties, thermal stability, antioxidant capacity, and pH‐responsive release, rendering it ideal for fruit preservation. In vivo experiments further confirmed its efficacy in maintaining the quality of strawberry (nonclimacteric) and kiwifruit (climacteric) over 8 d, delaying ripening and senescence while preserving their appearance, nutritional value, and antioxidant capacity. This study provides a novel strategy for designing high‐performance antimicrobial nanozymes and advances research on fruit preservation, offering significant potential for food packaging and preservation applications. The demonstrated photocatalytic coordination approach also shows promise as a general platform that may be extended to other fields such as biomedical (e.g., targeted therapy) and environmental catalysis (e.g., pollutant degradation). Future research should focus on optimizing its performance and exploring its applicability across different food systems to provide theoretical and technical support for advanced preservation technologies, while further investigating its broader potential.

## 4 Experimental Section

### Materials and Reagents

Zn(NO_3_)_2_·6H_2_O, 2‐methylimidazole, Cu(NO_3_)_2_·3H_2_O, sodium dicyanamide (DCDA), melamine (MA), α‐LA, and GSH were purchased from Aladdin Chemical Reagent Co., Ltd. (Shanghai, China). DCFH‐DA, SYTO9, propidium iodide (PI), OPDA, DTNB, TMB, DPBF, Gel, CS, glycerol (GL), HAc‐NaAc buffer, methanol, ethanol, acetic acid, isopropanol, DPPH, and ABTS were obtained from Sinopharm Chemical Reagent Co., Ltd. (Beijing, China). *E. coli* and *S. aureus* were purchased from Huankai Microorganism Co., Ltd. (Guangzhou, China). *B. cinerea* was acquired from Beijing Baio Bowei Biotechnology Co., Ltd. (Bio‐80834). Strawberries (“HongYan”) and kiwifruits (“Xuxiang”) were harvested from an orchard in Hefei, Anhui Province, China. All reagents were obtained from commercial sources and used without further purification.

### Synthesis of Cu/CNS via the Co‐Coordination of a Single Cu Site with N and S

The synthesis of Cu/CN began with the preparation of ZIF‐8 precursors by reacting Zn(NO_3_)_2_·6H_2_O and 2‐methylimidazole in methanol for 24 h. The precursors were pyrolyzed at 950 °C for 5 h in an inert atmosphere to produce NPC. Cu/CN was then fabricated by mixing NPC with Cu(NO_3_)_2_·3H_2_O, sodium dicyanamide, and melamine, followed by ultrasonication, stirring for 12 h, and heating at 650 °C for 2 h. The final product was treated with HCl to remove impurities.

Cu/CNS was synthesized by first dispersing 0.1 g of Cu/CN in 50 mL of distilled water. Varying amounts of α‐LA (0.05, 0.1, and 0.2 g) were dissolved in 200 mL of distilled water to achieve concentrations of 0.25, 0.5, and 1 g L^−1^,^[^ ^8^ ^]^ respectively. The α‐LA solutions were then added to the Cu/CN dispersion under magnetic stirring, followed by photocatalytic treatment using a UV high‐pressure mercury lamp (GGY250, China; 365 nm, 12.5 mW cm^−^^2^) for 2 h and additional stirring for 6 h in the dark. The resulting crystals (Cu/CNS‐0.25, Cu/CNS‐0.5, and Cu/CNS‐1) were collected by centrifugation, washed with ethanol, and vacuum‐dried at 80 °C for 12 h. NPC@LA was also prepared by dispersing 0.1 g of NPC in 50 mL of distilled water and adding 200 mL of α‐LA (1 g L^−1^) under magnetic stirring, following the same procedure as above. The resulting NPC@LA crystals were obtained after centrifugation, washing, and drying.

### Material Characterization

Based on the dose‐dependent responses of the prepared samples, Cu/CNS‐1 was selected for detailed characterization because it elicited a saturating effect with no further significant enhancements beyond the established threshold; Cu/CN was also characterized for comparison. The morphology, size, and elemental mapping of the samples were analyzed by TEM and high‐resolution TEM (Talos F200X G2, China). FTIR spectroscopy (PerkinElmer, USA) over the wavenumber range of 400–4000 cm^−1^ was used to identify functional group changes, and zeta potential measurements (Zetasizer Nano‐ZSE, England) were used to assess surface charge variations. The specific surface area and N_2_ adsorption–desorption isotherms of the synthesized materials were determined using the BET (Micromeritics ASAP 2460, USA) method, and pore size distributions were calculated using density functional theory (DFT). Phase structures were characterized by XRD (Rigaku MiniFlex600, Japan) with a step size of 0.05° over the 2θ range of 5°–60°. Chemical states and metal‐loading contents were analyzed using XPS (Thermo Scientific K‐Alpha, USA), Raman spectroscopy (Horiba LabRAM HR Evolution, Japan), and ICP‐OES (Agilent 7850, USA). AC‐HAADF‐STEM images were acquired using a JEM‐ARM200F TEM instrument (Japan) operated at 200 keV. XAFS, including the XANES and EXAFS regions of Cu (8979 eV), was performed at the SPring‐8 14b2 beamline using Si (111) crystals with the storage ring operating at 8.0 GeV and 99.5 mA electron currents. These analyses were conducted to provide comprehensive insights into the structural and chemical properties of Cu/CNS‐1 and Cu/CN.

### Safety Assessment

The biocompatibility of NPC, Cu/CN, and Cu/CNS‐1 was assessed using hemolysis assay and cytotoxicity evaluation. For the hemolysis assay, a 2% red blood cell suspension was mixed with sample solutions at concentrations ranging from 25 to 800 µg mL^−1^ and incubated at 37 °C for 12 h. The absorbance of the supernatant was measured at 492 nm, with sterile water and PBS as positive and negative controls, respectively. The hemolysis rate was calculated as follows:

(1) $$\mathrm{Hemolysis}\;\mathrm{rate}\;\left(\%\right)=\frac{\left(A_{1}-A_{0}\right)}{\left(A_{2}-A_{0}\right)}\times 100\%$$

where *A*_1_, *A*_2_, and *A*_0_ represent the absorbance values of the experimental group, positive control group, and negative control group, respectively.

Additionally, for the cytotoxicity evaluation, the cytocompatibility of NPC, Cu/CN, and Cu/CNS‐1 was assessed in RAW264.7 cells via a standard MTT assay. After seeding in 96‐well plates (1 × 10^5^ cells mL^−1^) for 12 h, the cells were incubated with the different materials at concentrations ranging from 25 to 800 µg mL^−1^ for 24 h. Subsequently, 10 µL of MTT solution was added to each well, and the plates were incubated for another 4 h. The resulting crystalline substance was then dissolved by adding 150 µL of dimethyl sulfoxide with gentle agitation for 10 min. The absorbance of each well was measured at 570 nm, using cells incubated in medium alone as the control.

### Enzyme‐Like Activity Assays

Determination of CAT‐like activity: The CAT‐like activity of Cu/CN and Cu/CNS was measured using UV‐vis spectroscopy. A 50 µg mL^−1^ material suspension was incubated with 1 mm H_2_O_2_ in PBS for 10 min, and H_2_O_2_ decomposition was monitored at 240 nm.

Determination of OXD‐like activity: OXD‐like activity was assessed using 3,3′,5,5′‐tetramethylbenzidine and o‐phenylenediamine as chromogenic probes. A 50 µg mL^−1^ material suspension was incubated with 1 mM TMB (or OPDA) in PBS for 10 min, and oxidation products were detected at 652 nm (TMB) or 414 nm (OPDA). •O_2_− generation was evaluated using the DPBF probe, with absorbance changes measured at 420 nm.

Validation of the CAT‐OXD cascade under endogenous H_2_O_2_ concentration: To verify the self‐sustaining catalytic cycle under physiologically relevant H_2_O_2_ levels (≈3 mM), two sets of experiments were performed using TMB as the chromogenic substrate. For ambient condition tests, three systems were compared: (i) Cu/CNS + TMB, (ii) Cu/CNS + TMB + 3 mM H_2_O_2_, and (iii) Cu/CNS + TMB + 3 mM H_2_O_2_ + 50 mM isopropanol (•OH scavenger). The chromogenic response was monitored at 652 nm after 10 min of reaction. For anaerobic environment tests, the same three systems were placed in a pure N_2_ atmosphere, and the absorbance at 652 nm was similarly measured to assess O_2_‐independent catalytic activity.

Kinetic analysis: Kinetic parameters were determined by incubating 50 µg/mL Cu/CN and Cu/CNS with varying TMB concentrations (0.1–1 mm). Initial reaction rates (*V*) were calculated using the Beer–Lambert law, and the data were fitted to the Michaelis–Menten equation.

(2) A=εbc

where *A* is the absorbance, *ε* is the molar extinction coefficient (L mol^−1^ cm^−1^), *b* is the path length (cm), and *c* is the concentration (mol L^−1^).

(3) $$V=\frac{\left(V_{\max}\times \left[S\right]\right)}{\left(K_{m}+\left[S\right]\right)}$$

where *V* is the initial reaction rate (µM/s), *V*_max_ is the maximum reaction rate (µM/s), [*S*] is the substrate concentration (mM), and *K*_m_ is the Michaelis–Menten constant. Catalytic activity (units) was calculated as follows:

(4) $$b=\frac{\left(V\times \Delta A\right)}{\left(\varepsilon \times l\times \Delta t\right)}$$

where *b* is the activity in units, *V* is the reaction volume, *ε* is the molar extinction coefficient, *l* is the path length, and Δ*A/*Δ*t* is the initial absorbance change at 652 nm. SA was derived as:

(5) $$a=\frac{b}{\left[m\right]}$$

where *a* is the SA in units per mg of material (U mg^−1^) and [*m*] is the mass of Cu/CN or Cu/CNS (mg).

Determination of GSH‐Ox‐like activity: GSH‐Ox‐like activity was evaluated using DTNB as the probe. A 500 µg mL^−1^ material suspension was incubated with 1 mM GSH in PBS for 10 min. The mixture was combined with DTNB, and absorbance was measured at 412 nm after 3 min.

### In Vitro Antimicrobial Capacity and Mechanism Evaluation

Colony counting assay: The antimicrobial efficacies of Cu/CN and Cu/CNS against *S. aureus*, *E. coli*, and *B. cinerea* were evaluated by colony counting. Bacterial (10^7^ CFU mL^−1^) or fungal (10^5^ CFU mL^−1^) suspensions were incubated with 2 mg mL^−1^ material suspensions in PBS for 3 h (bacteria = 37 °C, fungi = 28 °C). After dilution, the suspensions were spread onto agar plates and incubated (bacteria = overnight, fungi = 48 h). Inhibition rates were calculated as follows:

(6) $$\mathrm{Inhibition}\;\mathrm{rate}\;\left(\%\right)=\frac{N_{0}-N_{1}}{N_{0}}\times 100\%$$

where *N*_0_ and *N*_1_ represent the initial and post‐treatment colony counts, respectively. The controls included distilled water, NPC@LA, and α‐LA. All experiments were performed in triplicate.

Cell viability assay: Bacterial and fungal viability was assessed using the CCK‐8 assay. For bacteria, 200 µL of an incubated suspension was mixed with 20 µL of CCK‐8 solution and incubated at 37 °C for 1 h. For fungi, spore suspensions were first cultured in PDB medium at 28 °C for 72 h and then subjected to the same procedure described above. Absorbance was measured at 450 nm, and relative cell viability was calculated as follows:

(7) $$\mathrm{Relative}\;\mathrm{bacterial}\;\mathrm{vitality}\;\left(\%\right)=\frac{A_{2}-A_{0}}{A_{1}-A_{0}}\times 100\%$$

where *A*_0_, *A*_1_, and *A*_2_ represent the absorbance values of the blank control, distilled water treatment, and material treatment, respectively. All experiments were performed in triplicate.

Morphological observation of microorganisms: Microbial samples were fixed with 2.5% glutaraldehyde for 12 h, dehydrated using an ethanol gradient, and dispersed on carbon‐coated copper grids for SEM imaging (ZEISS Sigma 300, Germany).

DNA leakage assay: DNA leakage was determined using absorbance measurements at 260 nm. Bacterial suspensions were sampled at 0, 3, 6, 9, and 12 h, whereas fungal spore suspensions were cultured in PDB medium for 72 h before measurement. Samples were filtered through a 0.22 µm membrane prior to analysis.

ROS staining assay: ROS generation was detected using DCFH‐DA as the probe. The treated samples were stained with 25 µm DCFH‐DA for 30 min, incubated at 30 °C for 1 h, and observed under a fluorescence microscope (Olympus BX51, Tokyo, Japan) for green fluorescence.

Live/dead staining assay: Microbial viability was assessed using SYTO9 (live cells, green fluorescence) and PI (dead cells, red fluorescence). The samples were stained for 30 min, incubated at 30 °C for 1 h, and imaged under a confocal laser scanning microscope (Olympus FV3000, Japan).

Lipid peroxidation assays: The extent of lipid peroxidation in the bacteria and fungi cells following different treatments was evaluated according to our established protocols.^[^ ^8^ ^]^ The concentration of MDA, a key end‐product of lipid peroxidation, was quantified using the classic thiobarbituric acid reactive substances (TBARS) assay, and the absorbance was measured at 532 nm.

Key antioxidant enzyme activities: The activities of crucial antioxidant enzymes in the bacteria and fungi cells following different treatments were assessed according to our established methods.^[^ ^8^ ^]^ SOD activity was determined using the water‐soluble tetrazolium salt (WST‐1) method at 450 nm, which measured the enzyme's ability to inhibit the superoxide anion‐mediated reduction of WST‐1 to a water‐soluble formazan dye. CAT activity was quantified by directly monitoring the decomposition of H_2_O_2_ at 240 nm. POD activity was evaluated using guaiacol as a substrate by tracking the formation of the brown oxidative product at 470 nm. GSH‐Px activity was measured using the DTNB method, which monitored the decrease in reduced GSH concentration by tracking the absorbance of 2‐nitro‐5‐thiobenzoic acid (TNB) at 412 nm.

### Synthesis of Coating Films

Films were prepared using the solution‐casting method. Gel (5 g) was dissolved in 100 mL of acetic acid (1%, v/v) and then added with CS (100 mg) and GL (1.5 g, 30% (w/w) of the mass of Gel). Varying amounts of Cu/CNS‐1 (10–50 mg, corresponding to loadings of 100–500 µg mL^−1^) were incorporated into the mixture, which was subsequently stirred, ultrasonicated for 1 h, filtered, and homogenized at 5000 rpm for 3 min. After degassing, the solution was cast onto Petri dishes and dried at 45 °C for 12 h to produce films. The films were denoted as Cu‐CNS^x^@CS‐Gel, where x refers to the mass of Cu/CNS‐1. A control film without Cu/CNS‐1 was also prepared for comparison. All films were stored at 25 °C and 50% relative humidity for further analysis.

### Characterization of Coating Films

The surface morphology of the films was analyzed using SEM (EM30+, China) at 9.0 kV. Film thickness was measured at 10 random points using a handheld digital micrometer (Jingcheng, China), and the average value was recorded. Mechanical properties, including TS and EB, were evaluated using a texture analyzer (TA‐XTPLUS, UK). Moisture content was determined by weighing the films before (*W*_0_) and after drying at 75 °C for 24 h (*W*_1_) and then applying the following formula:

(8) $$\mathrm{Moisture}\;\mathrm{content}\;\left(\%\right)=\frac{W_{0}-W_{1}}{W_{0}}\times 100\%$$

where *W*_0_ (g) is the initial film weight and *W*_1_ (g) is the dry film weight.

WCAs were measured using a contact angle analyzer (Dataphysics OCA20, China) by depositing a 10 µL water droplet on the film surface. WVTR (g cm^−2^/24 h) were calculated by placing the films in a permeation cup containing 10 mL of distilled water and then recording the mass changes over 12 h.

(9) $$\mathrm{WVTR}=\frac{24\times \Delta m}{A\times t}$$

where Δ*m* (g) is the mass increment over time *t* (h) and *A* (cm^2^) is the area of the film exposed to water vapor transmission.

UV barrier properties were assessed using a UV‐vis spectrophotometer (Genesys 10S UV‐vis, USA) in the wavelength range of 200–800 nm. Functional group changes were analyzed using FTIR spectroscopy (PerkinElmer, USA) from 400 to 4000 cm^−1^. Thermal stability was evaluated using a simultaneous thermal analyzer (Netzsch TG 209 F3 Tarsus, Germany) by heating the films from 30 to 500 °C at a rate of 10 °C min^−1^ under N_2_. Antioxidant capacity was determined by measuring DPPH and ABTS radical scavenging activities. All experiments were performed in triplicate.

### pH‐Responsive Release Performance

Based on the results of comprehensive characterizations of the films, Cu/CNS^50^@CS‐Gel was selected to evaluate its pH‐responsive release behavior. The film (1 g) was immersed in 50 mL of HAc‐NaAc buffer solution at pH 5, 6, or 7 and incubated at room temperature (25 °C). At 12 h intervals, 5 mL of the solution was sampled, and its α‐LA content was quantified using UV‐vis spectrophotometry. The cumulative release of α‐LA was calculated using a standard calibration curve. The mixture was replenished with an equal volume of fresh buffer after each sampling, and the experiments were performed in triplicate to ensure reproducibility and accuracy.

### Metal Migration Test

The migration of copper ions from the Cu/CNS^50^@CS‐Gel film was quantified using ICP‐OES (Agilent 7850, USA). Specifically, the film was immersed in 100 mL of 10% ethanol solution in a sealed sterile beaker. Leachate samples were collected at 0, 2, 4, 6, and 8 days. After each sampling, a corresponding volume of deionized water was replenished to maintain the total volume. Each 1 mL sample was mixed with 0.5 mL of aqua regia, heated until complete dissolution, and then diluted to a final volume of 10 mL for analysis.

### Application of the Coating Film to Fruit Preservation

Freshly harvested strawberries (non‐climacteric) and kiwifruits (climacteric) were divided into three groups, immersed in distilled water, CS‐Gel film solution, or Cu/CNS^50^@CS‐Gel film solution for 3 min, and dipped for 2 s in CaCl_2_ solution. The fruits were stored at 25 °C and 75% relative humidity. Samples were collected on days 0, 2, 4, 6, and 8 to assess their L*/ a* value, flesh firmness, weight loss, decay rate, total soluble solid (TSS) content, ethylene production, and microbial indicators. To further substantiate the safety of the coated fruits, strawberries (pulp) and kiwifruits (both peel and pulp) samples collected on day 0 and day 8 of storage were analyzed by ICP‐OES to determine potential copper ion migration. Optical photographs were taken to document physical changes. All experiments were performed in triplicate to ensure reproducibility.

The antioxidant capacity and nutritional quality of the fruits were evaluated by measuring total phenolic content, total flavonoid content, MDA levels, H_2_O_2_ content, DPPH and ABTS radical scavenging activities, and the activities of POD, SOD, and CAT. Additionally, the levels of ascorbic acid (ASA), dehydroascorbic acid (DHA), GSH, and GSSG were quantified, with the ASA/DHA and GSH/GSSG ratios used to assess the regulatory effects of the composite coatings on nutritional quality. All experiments were performed in triplicate to ensure accuracy.

### Statistical Analysis

Performed one‐way analysis of variance (ANOVA) using SPSS 20.0. The results were expressed as mean ± standard deviation (SD). All experiments were conducted independently at least three times. The unpaired Student's bilateral *t*‐test was used to assess the significance of the data: ^*^ *p*<0.05, ^**^ *p*<0.01, ^***^ *p* <0.001. Means followed by different letters are significantly different at *p* < 0.05.

## Conflict of Interest

The authors declare no conflict of interest.

## Author Contributions

C.M. and C.W. contributed equally to this work. C.M. wrote original draft, performed methodology, investigation, acquired funding acquisition, performed data curation, conceptualization; C.W. wrote original draft, performed methodology, investigation, formal analysis, data curation, conceptualization; L.W. performed software, methodology; S.G. performed software, methodology; Y.M. performed investigation, methodology, wrote, reviewed, and edited the draft; W.L. performed investigation, methodology, wrote, reviewed, and edited the original manuscript; C.L. performed conceptualization, wrote, reviewed, and edited the manuscript; L.Z. collected resources, performed supervision, project administration, wrote, reviewed, edited the original manuscript.

## Supporting information



Supporting Information

## Data Availability

The data that support the findings of this study are available from the corresponding author upon reasonable request.
