# Supplementary material for: Photocatalytically Activated Cu‐N1S3 Single‐Atom Nanozyme: Enhancing Enzyme Activities and Antibacterial Synergy for Highly Efficient Fruit Preservation
Source: Adv Sci (Weinh). 2025 Dec 12;13(12):e15542. doi: 10.1002/advs.202515542 (PMC12948287; doi:10.1002/advs.202515542)
Supplement: Supplementary file 1 — Supporting Information [file ADVS-13-e15542-s001.docx]

**Supplementary Information**

**Photocatalytically Activated Cu-N_1_S_3_ Single-Atom Nanozyme: Enhancing Enzyme Activities and Antibacterial Synergy for Highly Efficient Fruit Preservation**

Chuanlong Men ^a, 1^, Chenchen Wu ^a, 1^, Lei Wang ^a^, Shengjie Gao ^a^, Yu Mao ^a^, Wei Liu ^a^, Changhong Liu ^a*^, Lei Zheng ^a*^

^a^ Engineering Research Center of Bio-Process, Ministry of Education, School of Food and Biological Engineering, Hefei University of Technology, Hefei 230009, China

^1^ These authors contributed equally to this work.

* Corresponding authors: Address: School of Food and Biological Engineering, Hefei University of Technology, Hefei, 230009, China. Tel.: +86 551 62901516.

E-mail addresses: changhong22@hfut.edu.cn (C. Liu); lzheng@hfut.edu.cn, lei.zheng@aliyun.com (L. Zheng)

**Result Section**





**Fig. S1.** FTIR spectra of Cu/CN, Cu/CNS-1 and LA.





**Fig. S2.** Zeta potentials of Cu/CN and Cu/CNS-1. The unpaired Student's bilateral t-test was used to assess the significance of the data: **P*<0.05, ***P*<0.01, ****P* <0.001.





**Fig. S3.** BET of Cu/CN and Cu/CNS-1.





**Fig. S4.** Pore diameter of Cu/CN and Cu/CNS-1.





**Fig. S5.** Surface area of Cu/CN and Cu/CNS-1. The unpaired Student's bilateral t-test was used to assess the significance of the data: **P*<0.05, ***P*<0.01, ****P* <0.001.


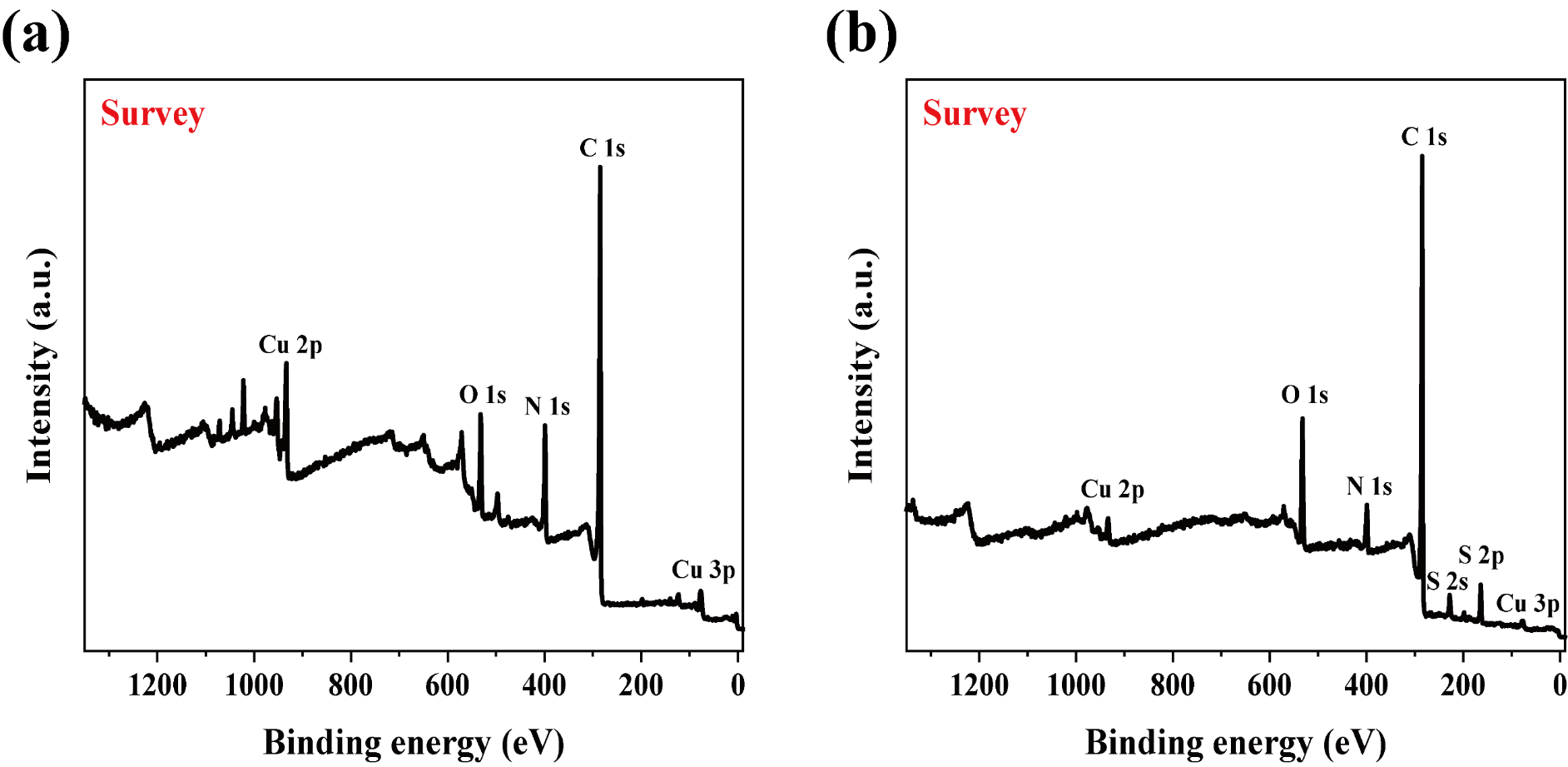


**Fig. S6.** XPS spectra of Cu/CN (a) and Cu/CNS-1 (b).





**Fig. S7.** XPS spectra of Cu orbitals in Cu/CN.


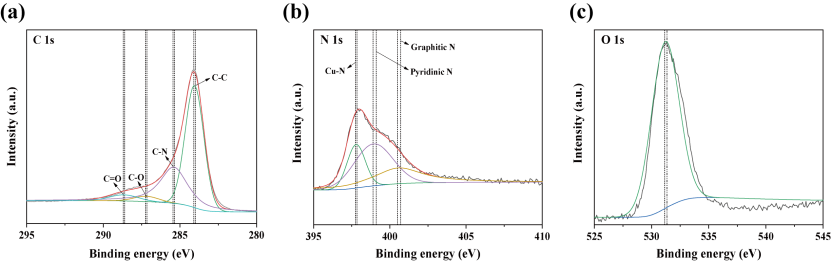


**Fig. S8.** XPS spectra of C orbitals (a), N orbitals (b), and O orbitals (c) in Cu/CN and Cu/CNS-1.

**
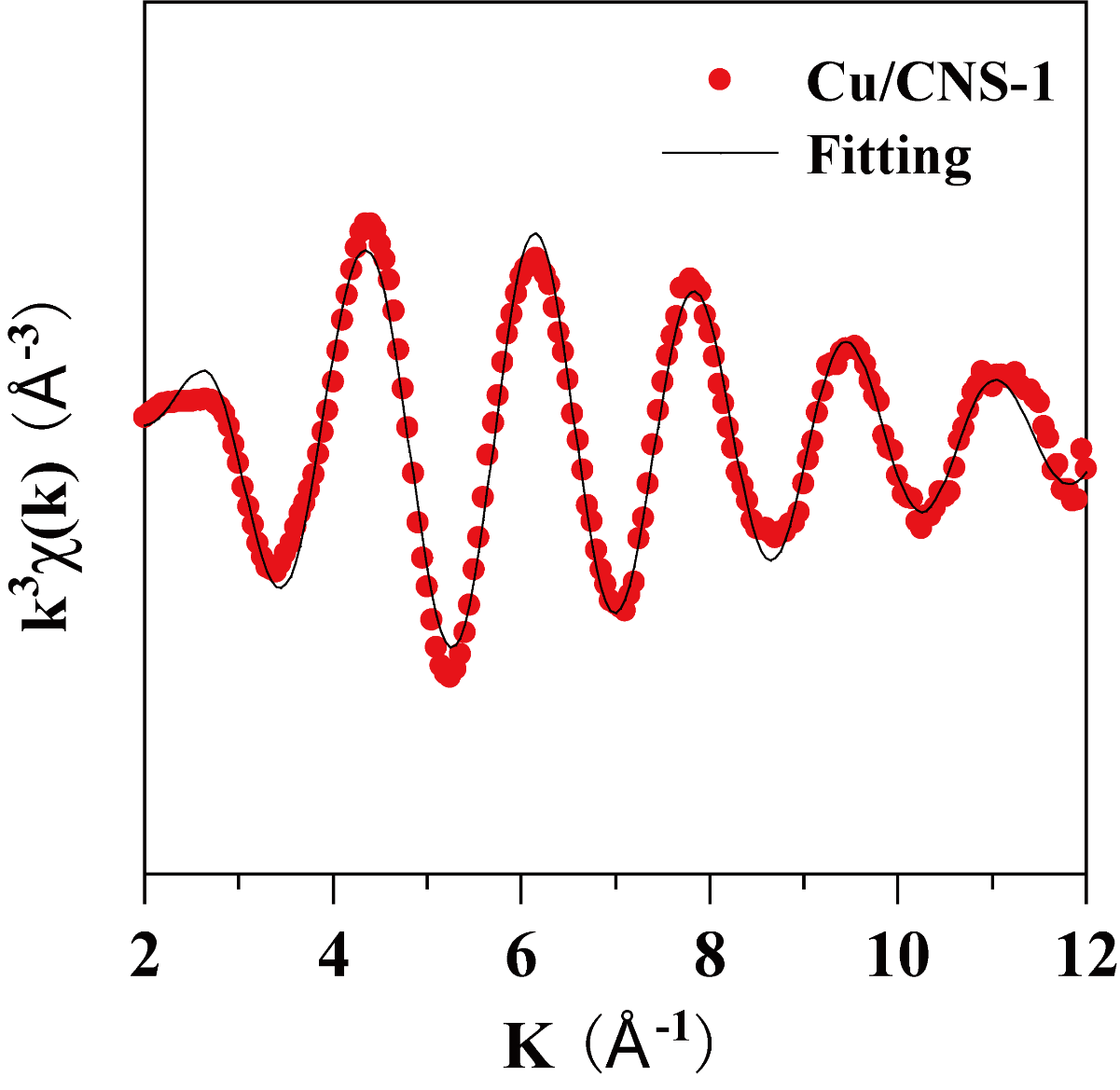
**

**Fig. S9.** EXAFS fitting result of Cu/CNS-1 at k space.

**
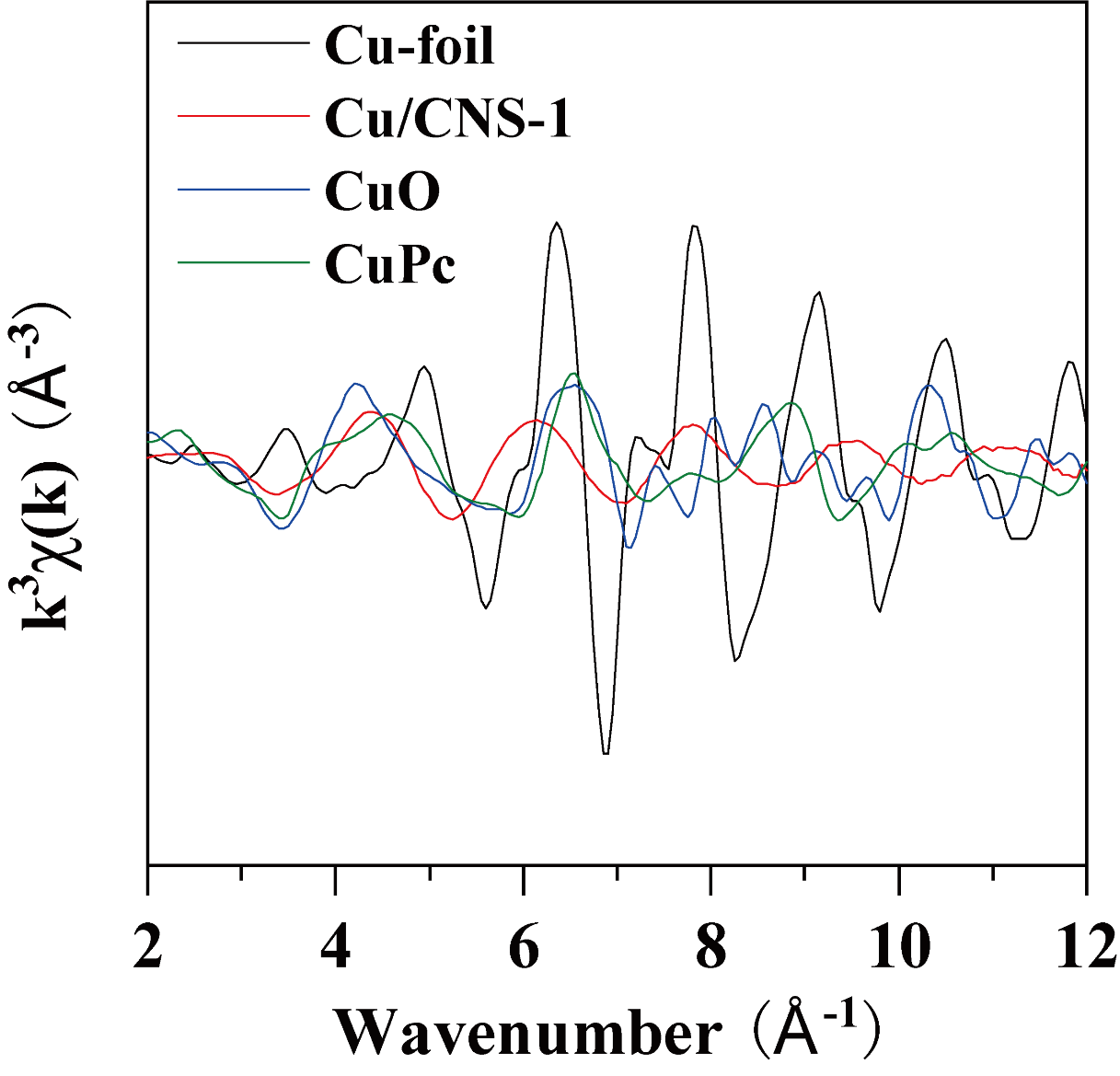
**

**Fig. S10.** Fourier-transform of the k^3^-weighted EXAFS spectra at Cu K-edge for Cu/CNS-1 (the fitting curves in K space).


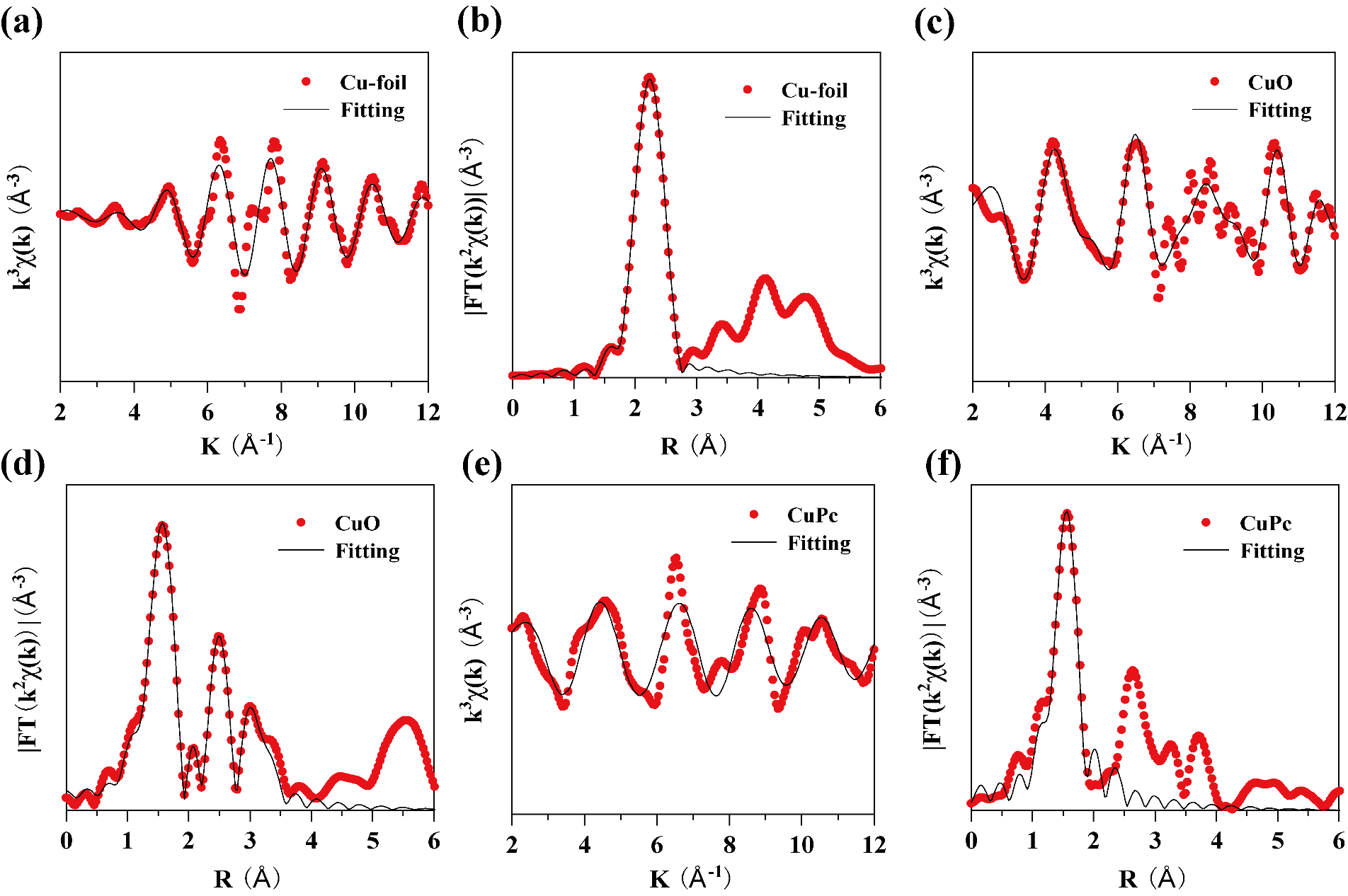


**Fig. S11.** EXAFS fitting results of Cu-foil (a), CuO (c) and CuPc (e) at k space. EXAFS fitting results of Cu-foil (b), CuO (d) and CuPc (f) in R space.


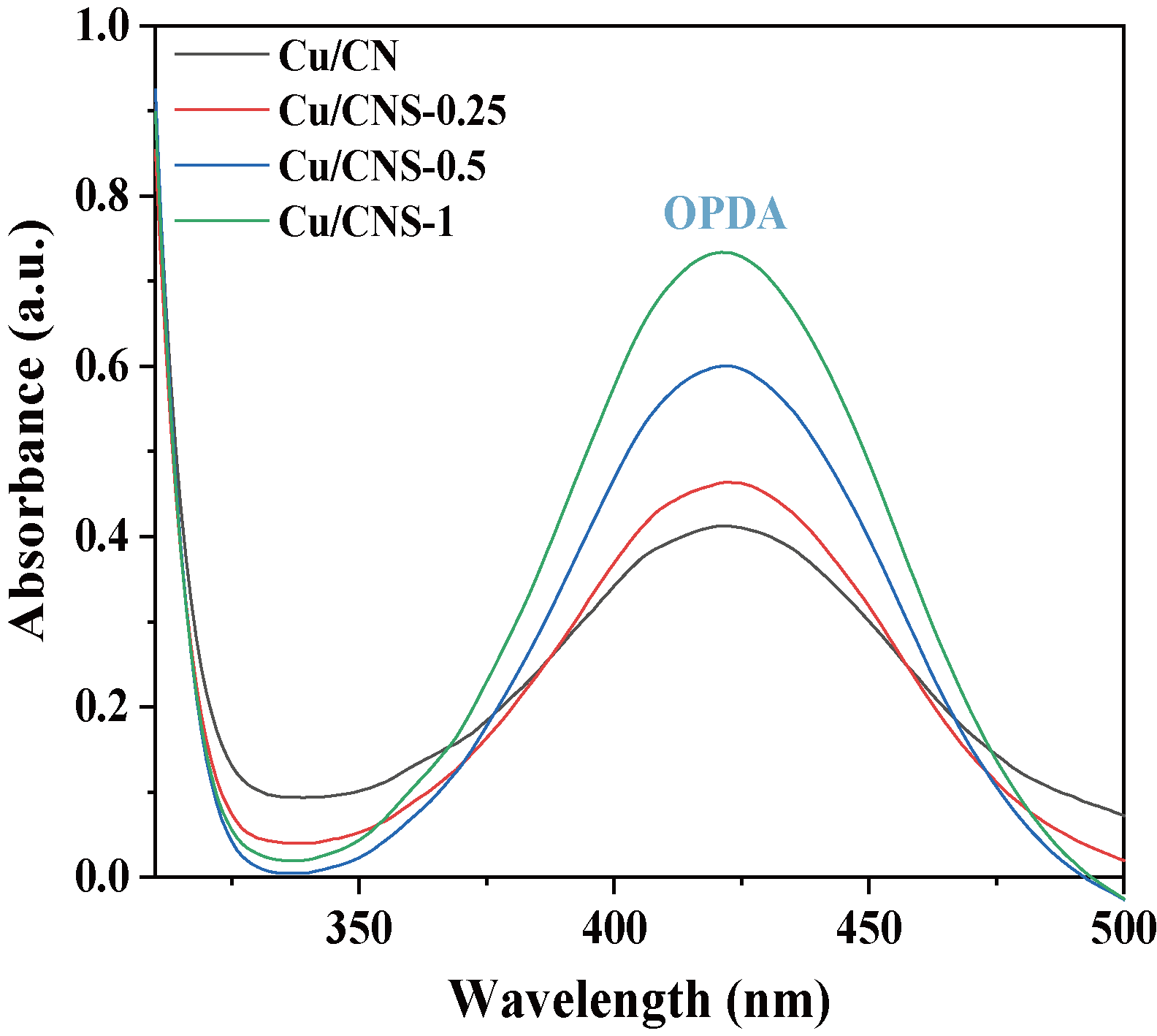


**Fig. S12.** Time-dependent absorption changes of OPDA in the presence of Cu/CN, Cu/CNS-0.25, Cu/CNS-0.5 and Cu/CNS-1.





**Fig. S13.** Time-dependent absorption changes of DPBF in the presence of Cu/CN, Cu/CNS-0.25, Cu/CNS-0.5 and Cu/CNS-1.


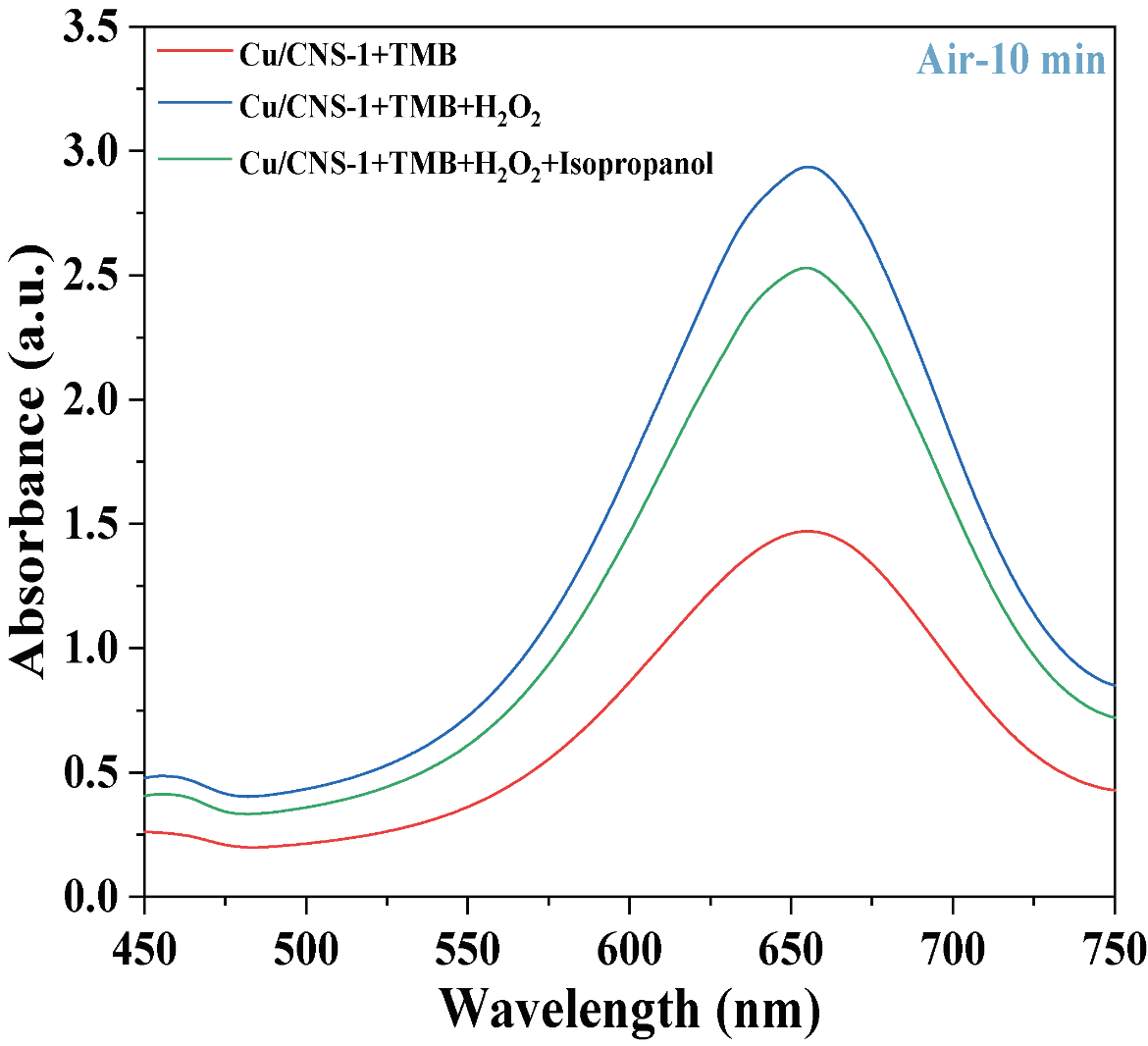


**Fig. S14.** The absorption value changes of TMB for the systems: Cu/CNS-1 + TMB, Cu/CNS-1 + TMB + H_2_O_2_, and Cu/CNS-1 + TMB + H_2_O_2_ + Isopropanol in air.


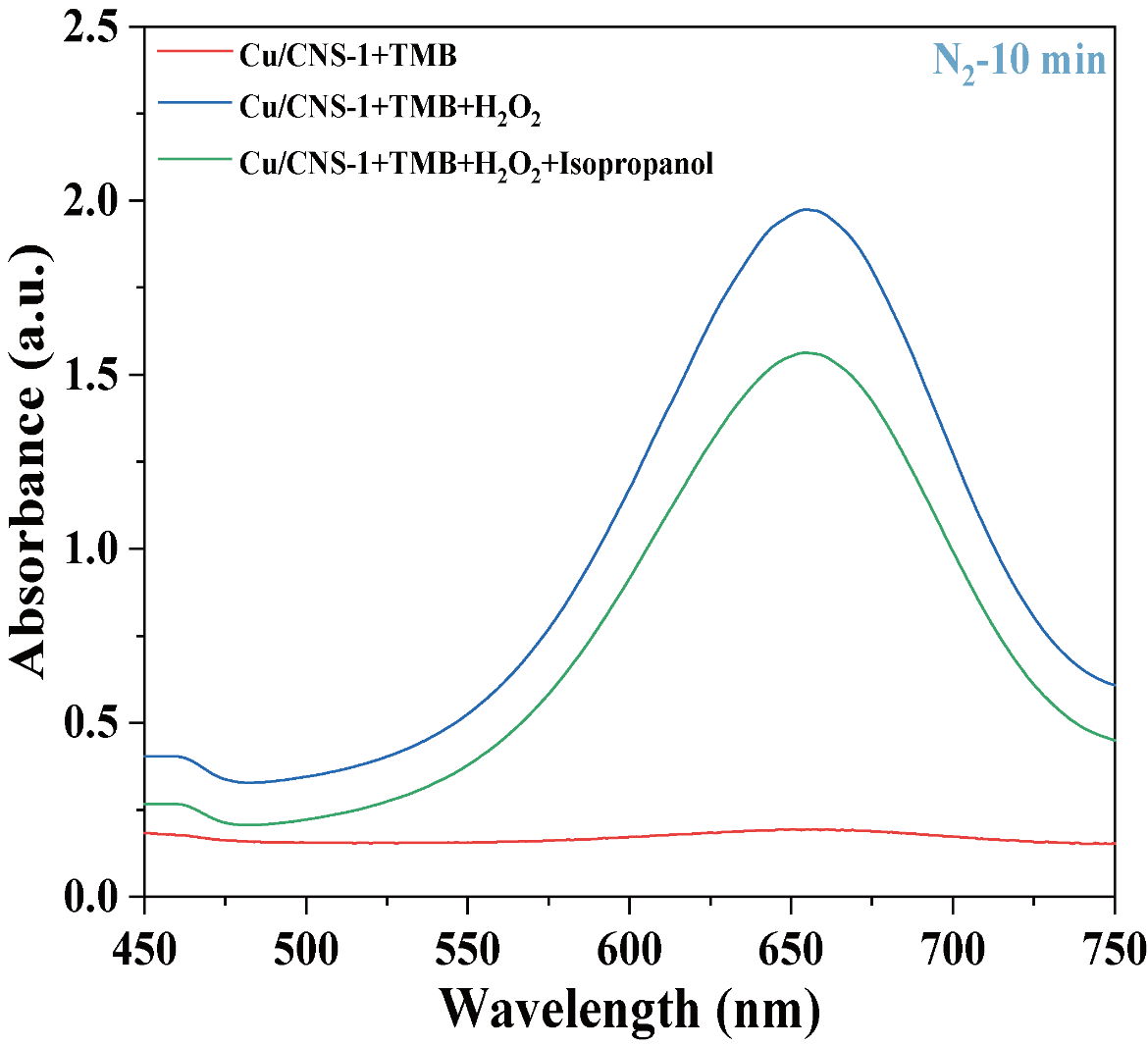


**Fig. S15.** The absorption value changes of TMB for the systems: Cu/CNS-1 + TMB, Cu/CNS-1 + TMB + H_2_O_2_, and Cu/CNS-1 + TMB + H_2_O_2_ + Isopropanol in N_2_.


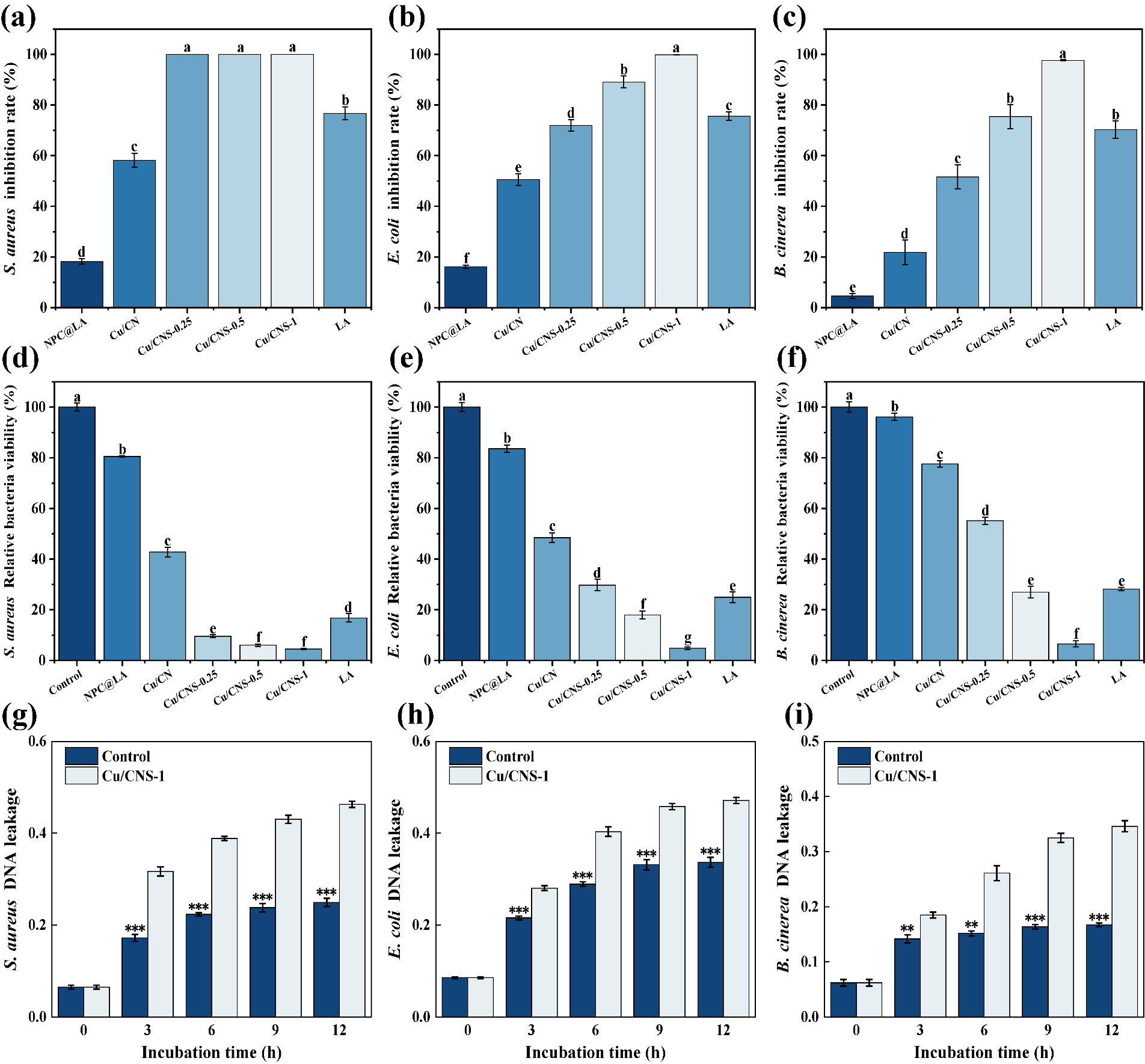


**Fig. S16.** Inhibition rates for *S. aureus* (a), *E. coli* (b) and *B. cinerea* (c) of NPC@LA, Cu/CN, Cu/CNS-0.25, Cu/CNS-0.5, Cu/CNS-1 and LA. Relative bacterial vitality for *S. aureus* (d), *E. coli* (e) and *B. cinerea* (f) of control, NPC@LA, Cu/CN, Cu/CNS-0.25, Cu/CNS-0.5, Cu/CNS-1 and LA. DNA leakage for *S. aureus* (g), *E. coli* (h) and *B. cinerea* (i) of control and Cu/CNS-1. The unpaired Student's bilateral t-test was used to assess the significance of the data: **P*<0.05, ***P*<0.01, ****P* <0.001. Means followed by different letters are significantly different at *P* < 0.05.


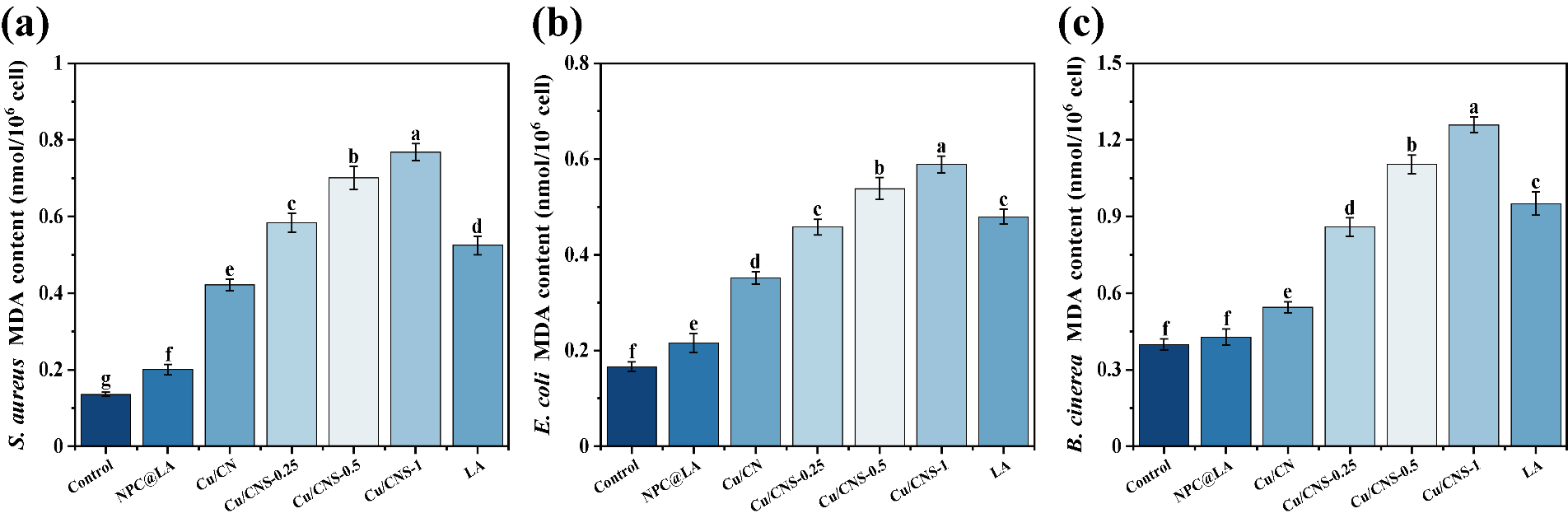


**Fig. S17.** MDA content for *S. aureus* (a), *E. coli* (b), and *B. cinerea* (c) of control, NPC@LA, Cu/CN, Cu/CNS-0.25, Cu/CNS-0.5, Cu/CNS-1 and LA. Means followed by different letters are significantly different at *P* < 0.05.


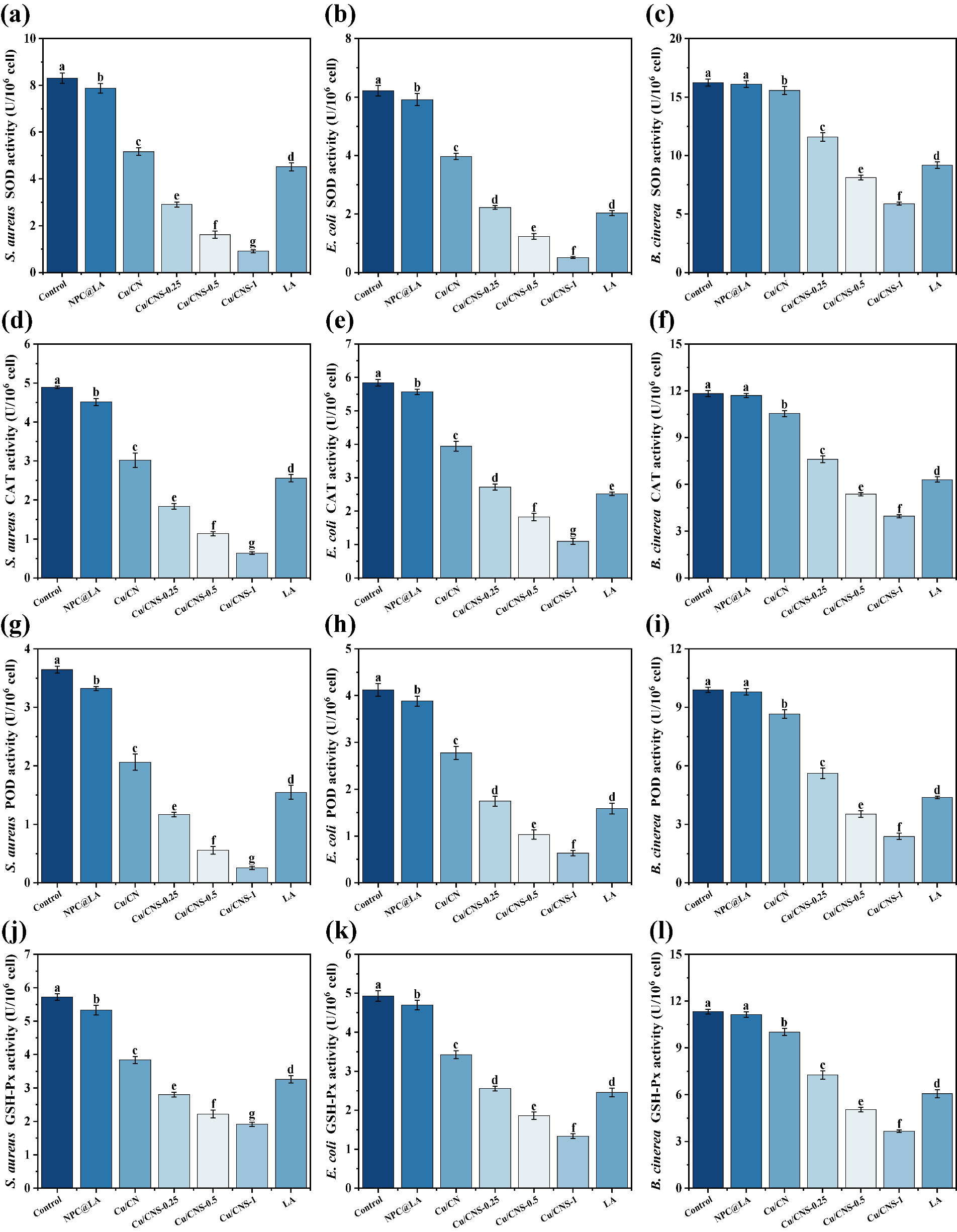


**Fig. S18.** SOD, CAT, POD, and GSH-Px activities for *S. aureus* (a, d, g, and j), *E. coli* (b, e, h, and k), and *B. cinerea* (c, f, i, and l) of control, NPC@LA, Cu/CN, Cu/CNS-0.25, Cu/CNS-0.5, Cu/CNS-1, and LA. Means followed by different letters are significantly different at *P* < 0.05.





**Fig. S19.** Steady-state kinetics of Cu/CNS-1.





**Fig. S20.** Specific activities of Cu/CNS-1 for substrate TMB.


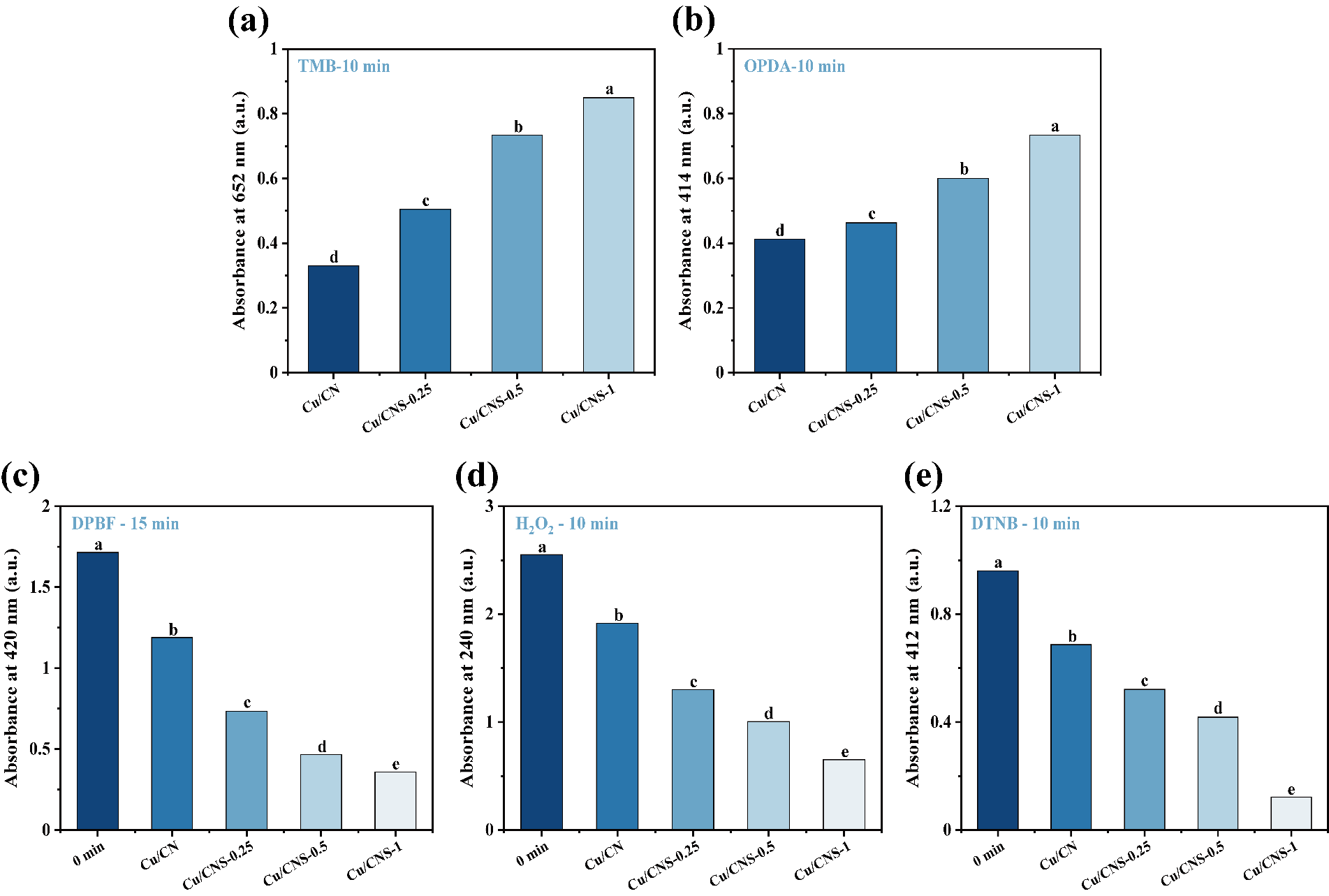


**Fig. S21.** Time-dependent absorption values changes of TMB (a), OPDA (b), DPBF (c), H_2_O_2_ (d), and DTNB (e) in the presence of Cu/CN, Cu/CNS-0.25, Cu/CNS-0.5 and Cu/CNS-1. Means followed by different letters are significantly different at *P* < 0.05.

**Fig. S22.** FTIR of CS-Gel film, Cu/CNS^10^@CS-Gel film, Cu/CNS^20^@CS-Gel film, Cu/CNS^30^@CS-Gel film, Cu/CNS^40^@CS-Gel film and Cu/CNS^50^@CS-Gel film.


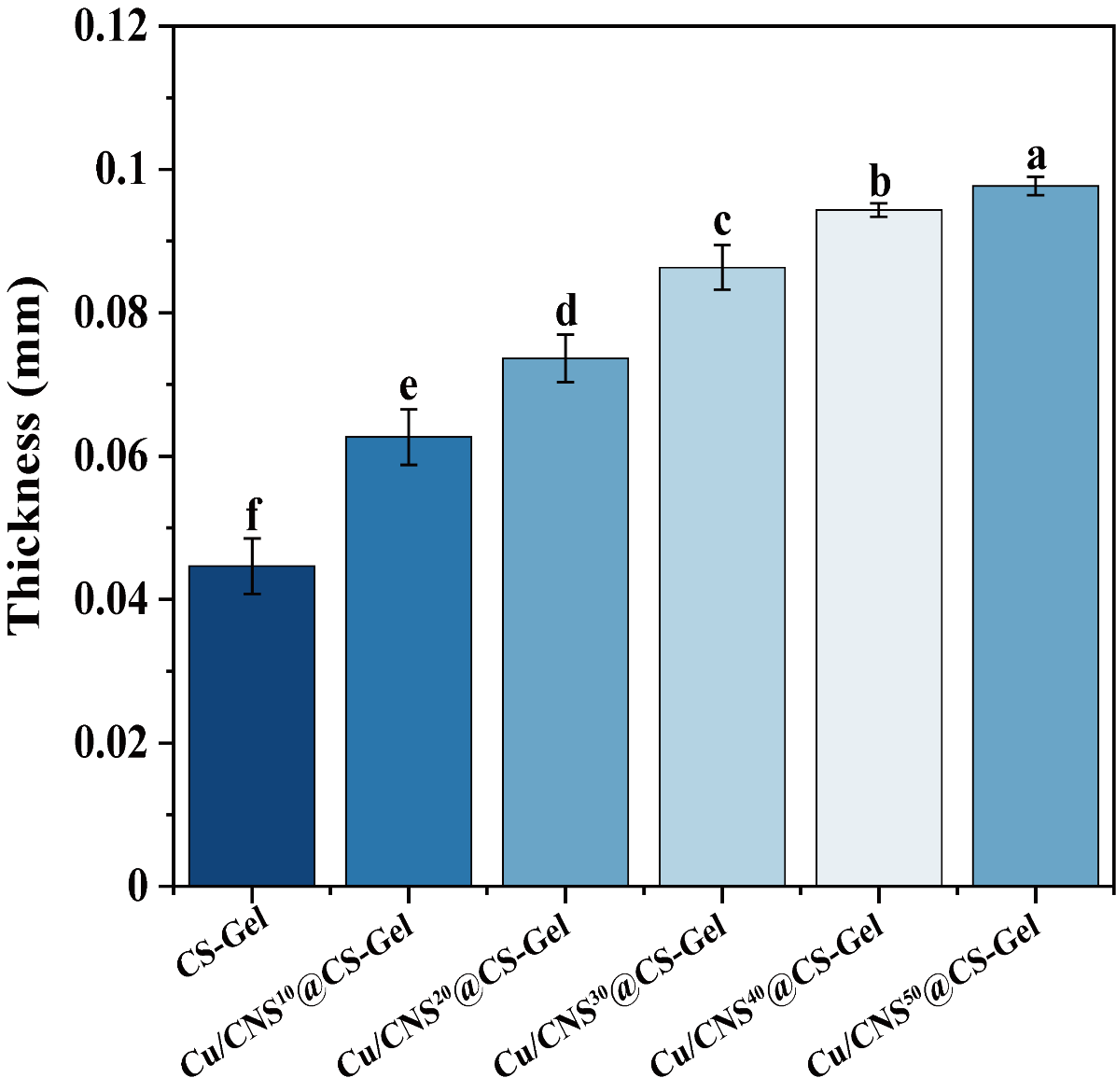


**Fig. S23.** Thickness of CS-Gel film, Cu/CNS^10^@CS-Gel film, Cu/CNS^20^@CS-Gel film, Cu/CNS^30^@CS-Gel film, Cu/CNS^40^@CS-Gel film and Cu/CNS^50^@CS-Gel film. Means followed by different letters are significantly different at *P* < 0.05.


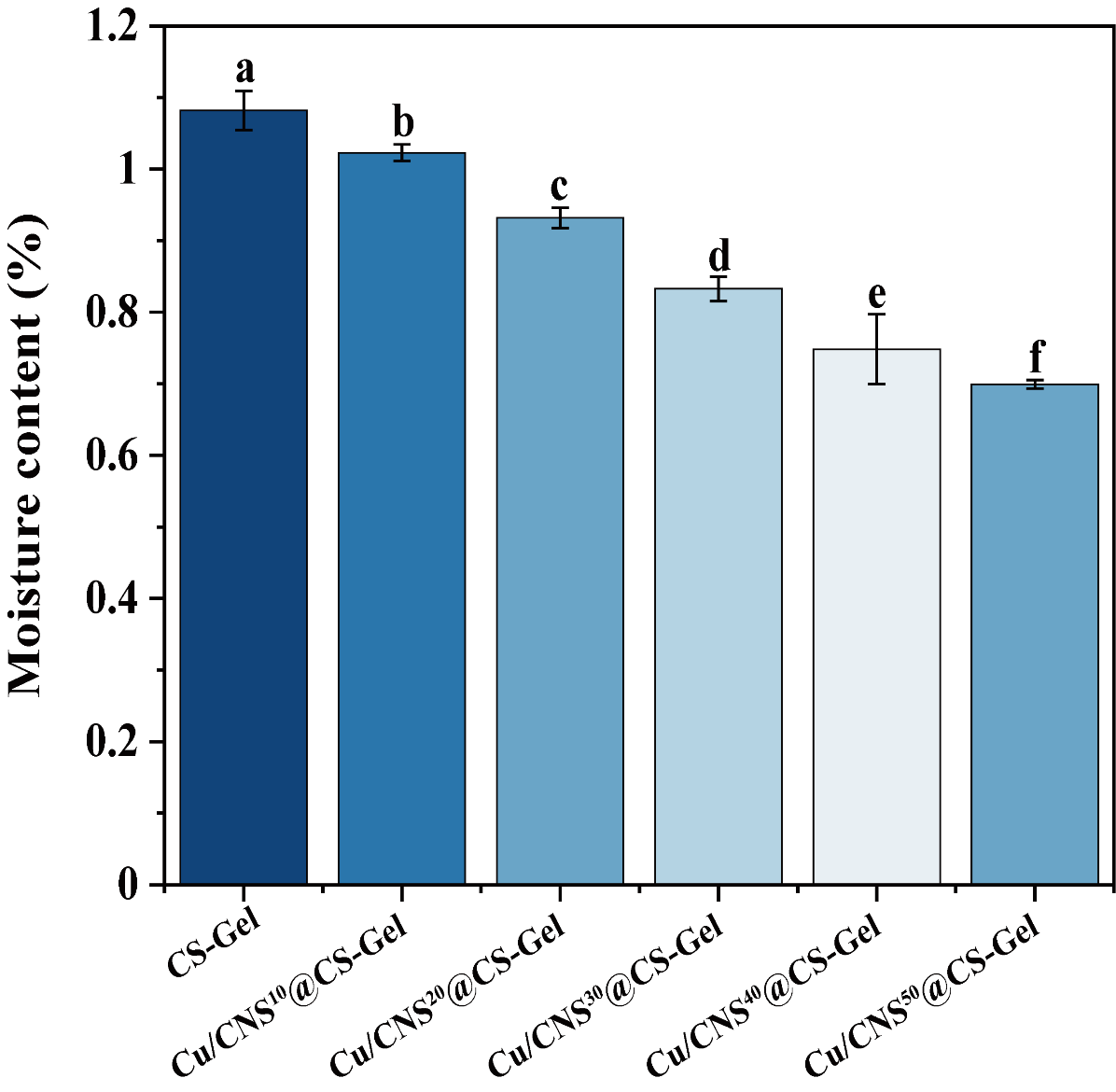


**Fig. S24.** Moisture content of CS-Gel film, Cu/CNS^10^@CS-Gel film, Cu/CNS^20^@CS-Gel film, Cu/CNS^30^@CS-Gel film, Cu/CNS^40^@CS-Gel film and Cu/CNS^50^@CS-Gel film. Means followed by different letters are significantly different at *P* < 0.05.

**Fig. S25.** DTG of CS-Gel film, Cu/CNS^10^@CS-Gel film, Cu/CNS^20^@CS-Gel film, Cu/CNS^30^@CS-Gel film, Cu/CNS^40^@CS-Gel film and Cu/CNS^50^@CS-Gel film.





**Fig. S26.** TGA of CS-Gel film, Cu/CNS^10^@CS-Gel film, Cu/CNS^20^@CS-Gel film, Cu/CNS^30^@CS-Gel film, Cu/CNS^40^@CS-Gel film and Cu/CNS^50^@CS-Gel film.


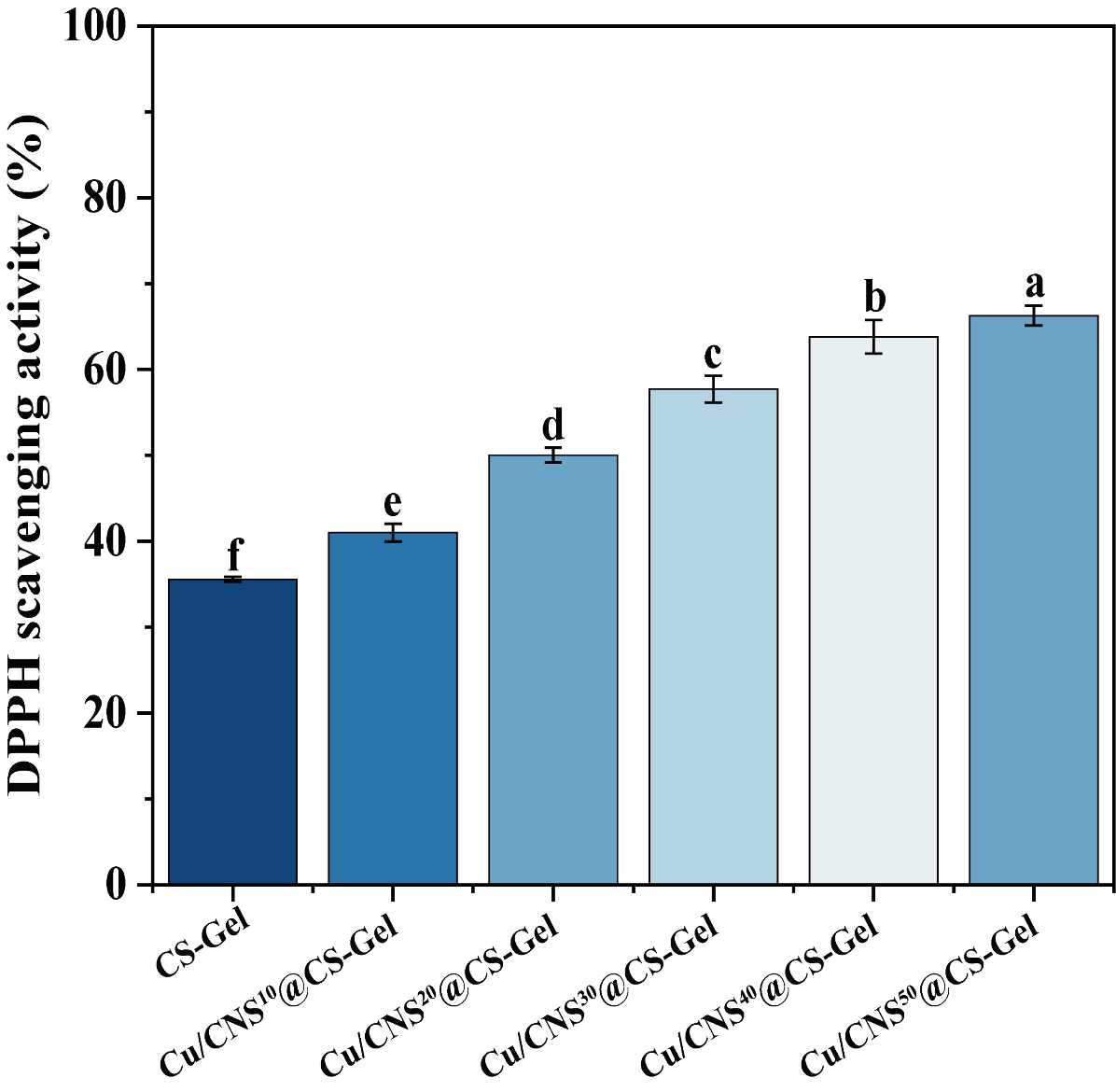


**Fig. S27.** DPPH scavenging activity of CS-Gel film, Cu/CNS^10^@CS-Gel film, Cu/CNS^20^@CS-Gel film, Cu/CNS^30^@CS-Gel film, Cu/CNS^40^@CS-Gel film and Cu/CNS^50^@CS-Gel film. Means followed by different letters are significantly different at *P* < 0.05.


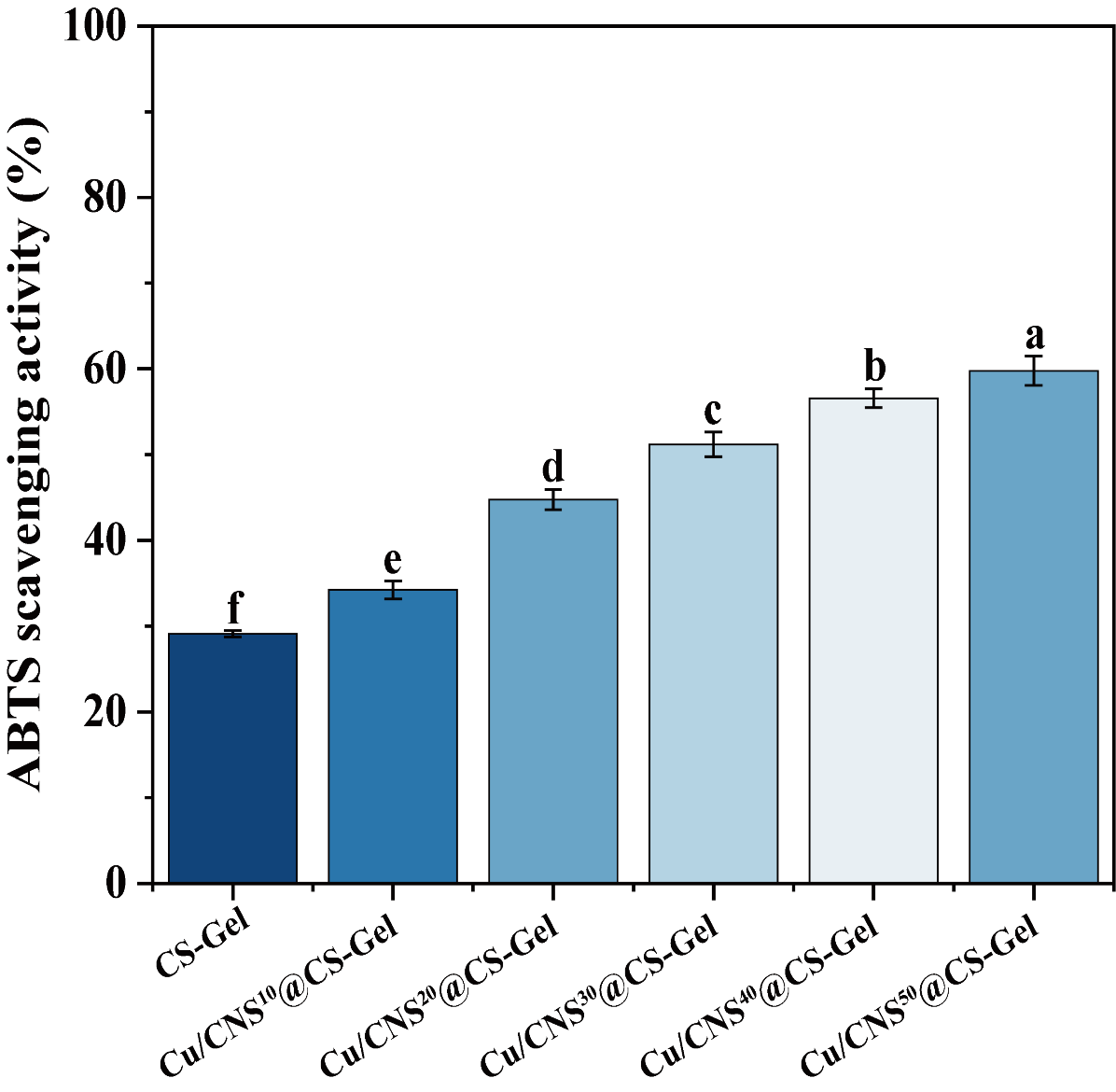


**Fig. S28.** ABTS scavenging activity of CS-Gel film, Cu/CNS^10^@CS-Gel film, Cu/CNS^20^@CS-Gel film, Cu/CNS^30^@CS-Gel film, Cu/CNS^40^@CS-Gel film and Cu/CNS^50^@CS-Gel film. Means followed by different letters are significantly different at *P* < 0.05.


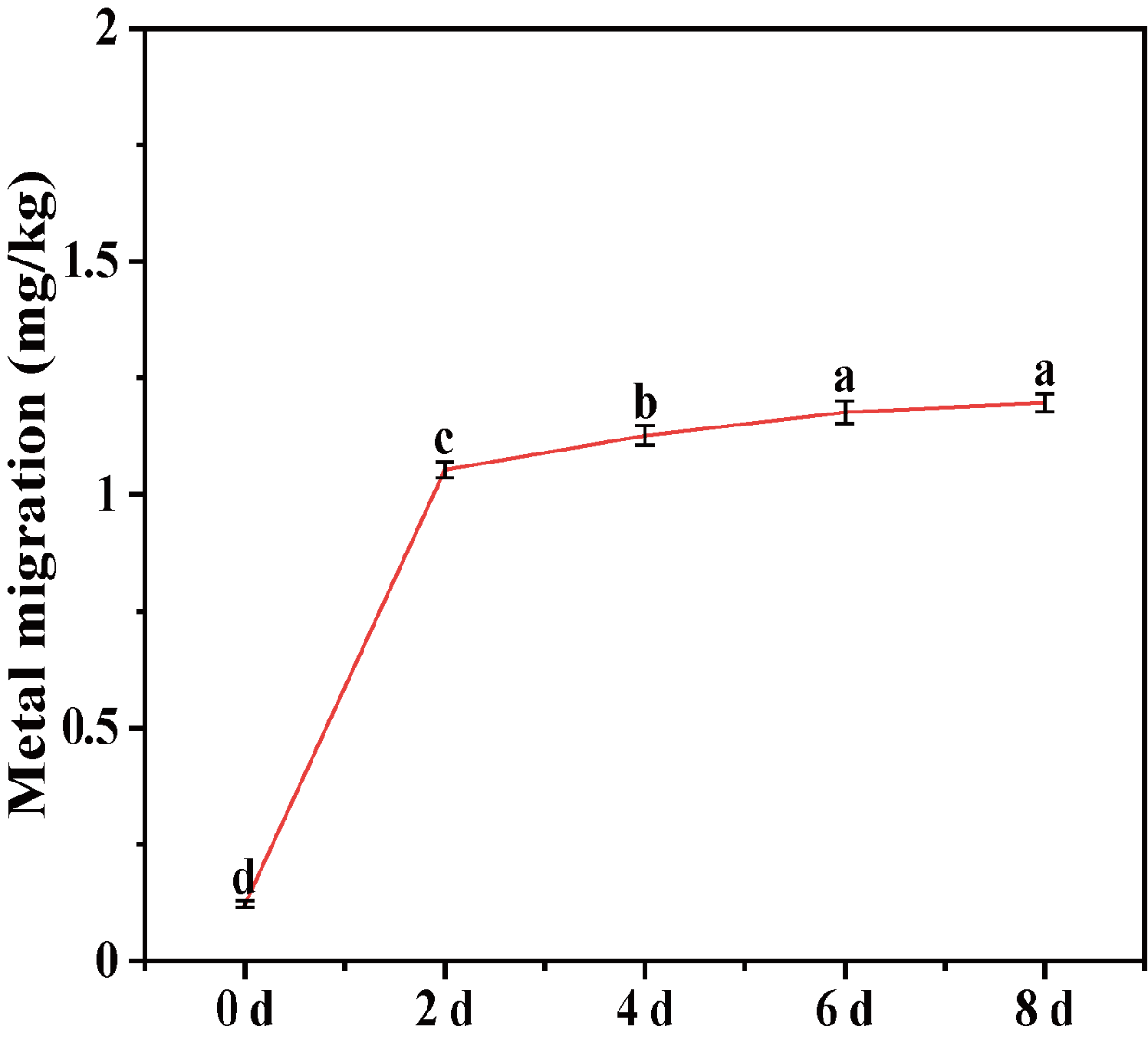


**Fig. S29.** Migration of Cu element from Cu/CNS^50^@CS-Gel film on days 0, 2, 4, 6, and 8. Means followed by different letters are significantly different at *P* < 0.05.


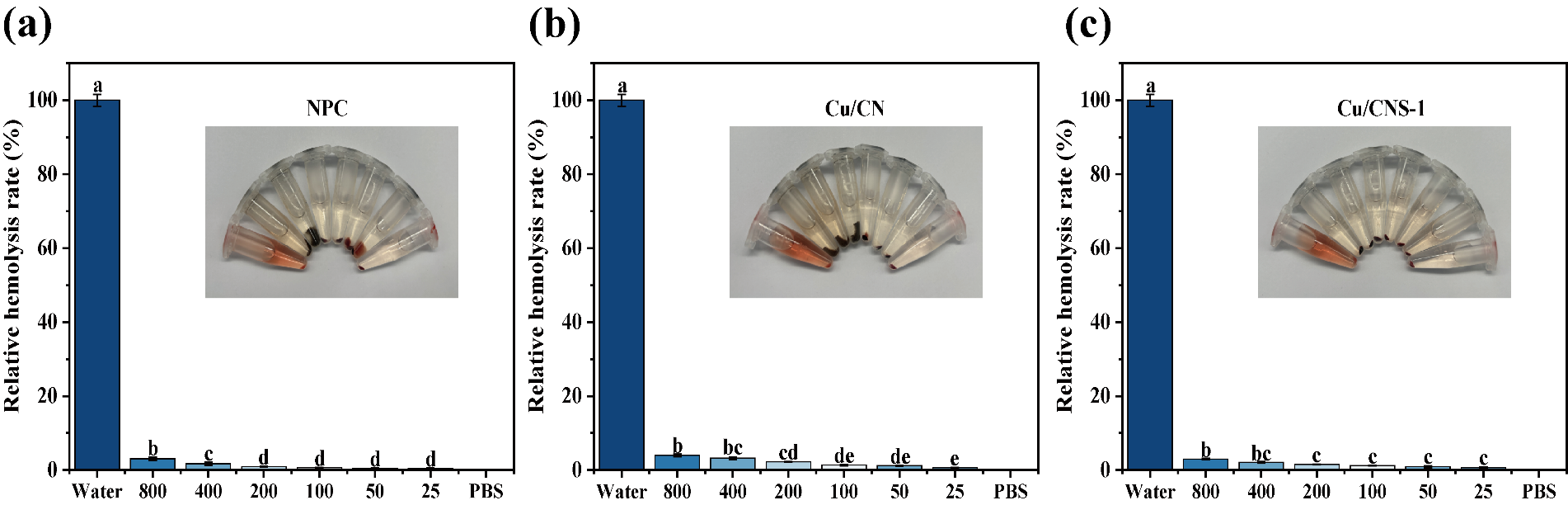


**Fig. S30.** Hemolysis rate of NPC (a), Cu/CN (b) and Cu/CNS-1 (c). Means followed by different letters are significantly different at *P* < 0.05.


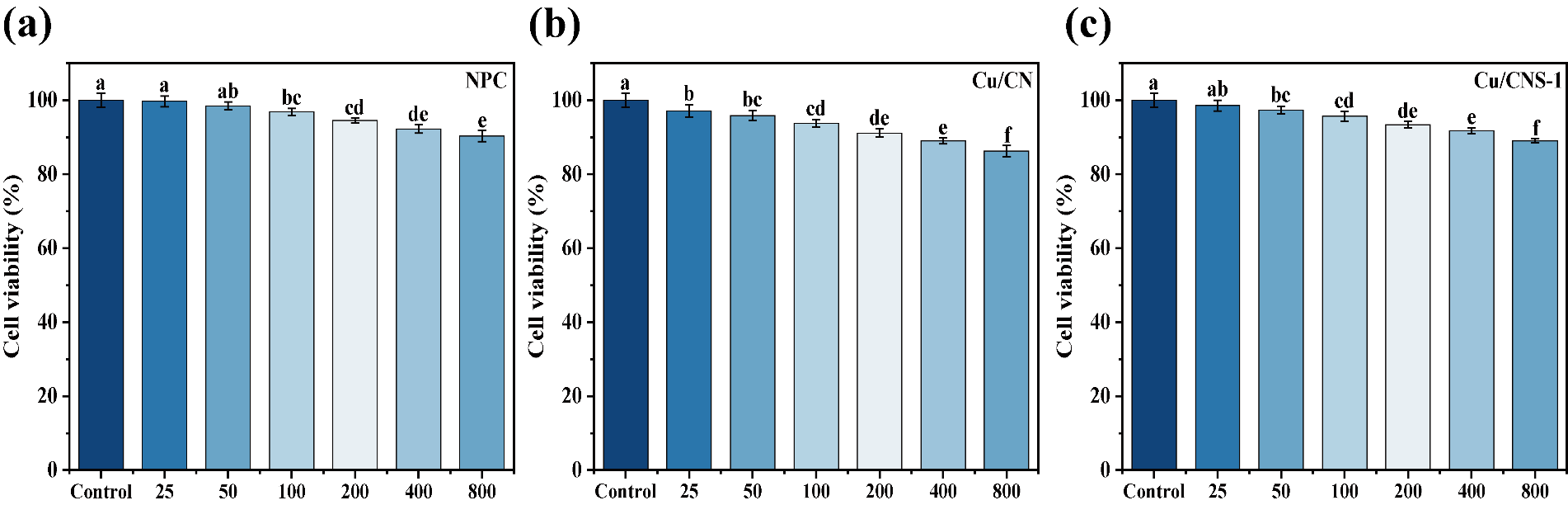


**Fig. S31.** Cytotoxicity of NPC (a), Cu/CN (b) and Cu/CNS-1 (c) against RAW264.7 cells *in vitro*. Means followed by different letters are significantly different at *P* < 0.05.


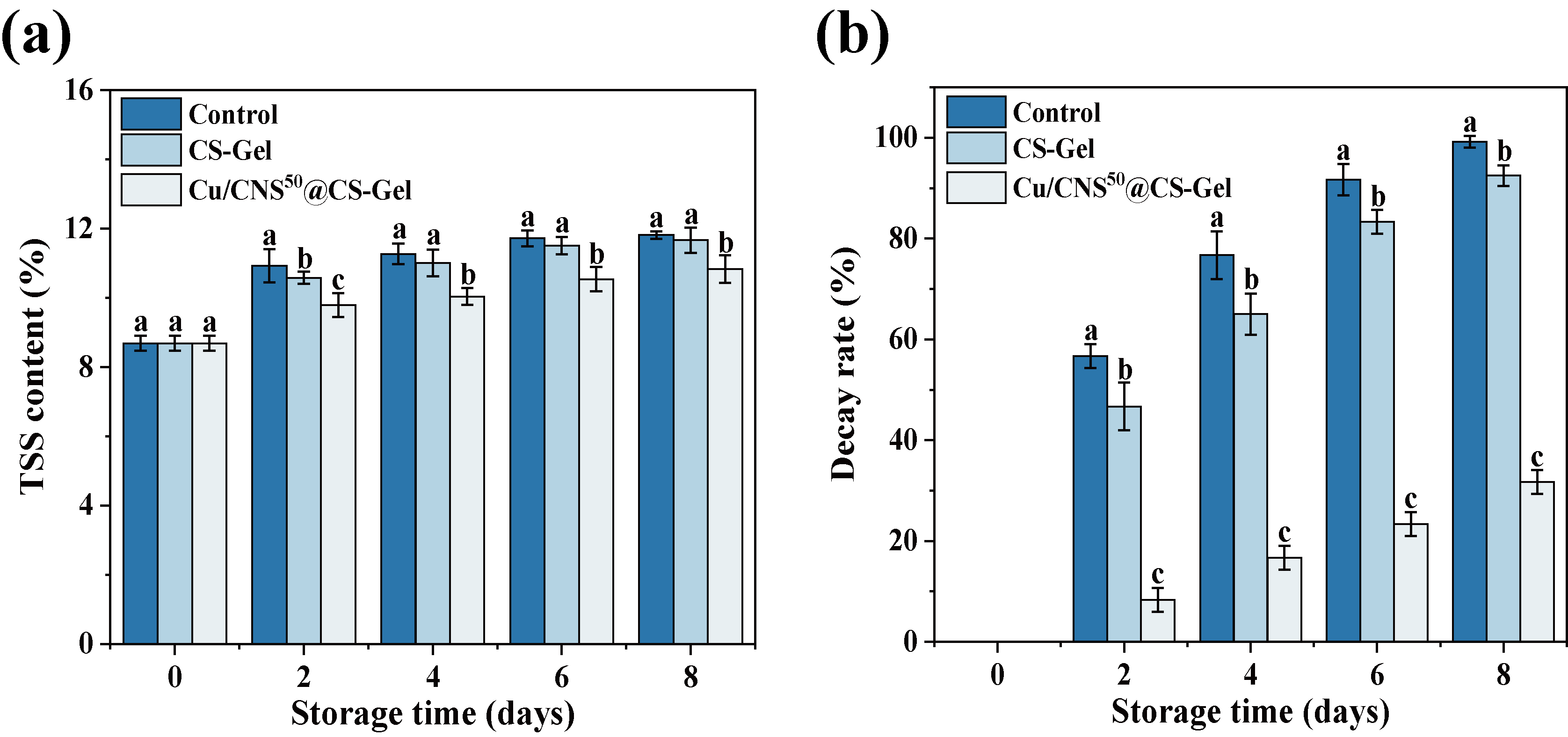


**Fig. S32.** TSS content (a), and decay rate (b) of postharvest strawberries (non-climacteric fruit). Means followed by different letters are significantly different at *P* < 0.05.


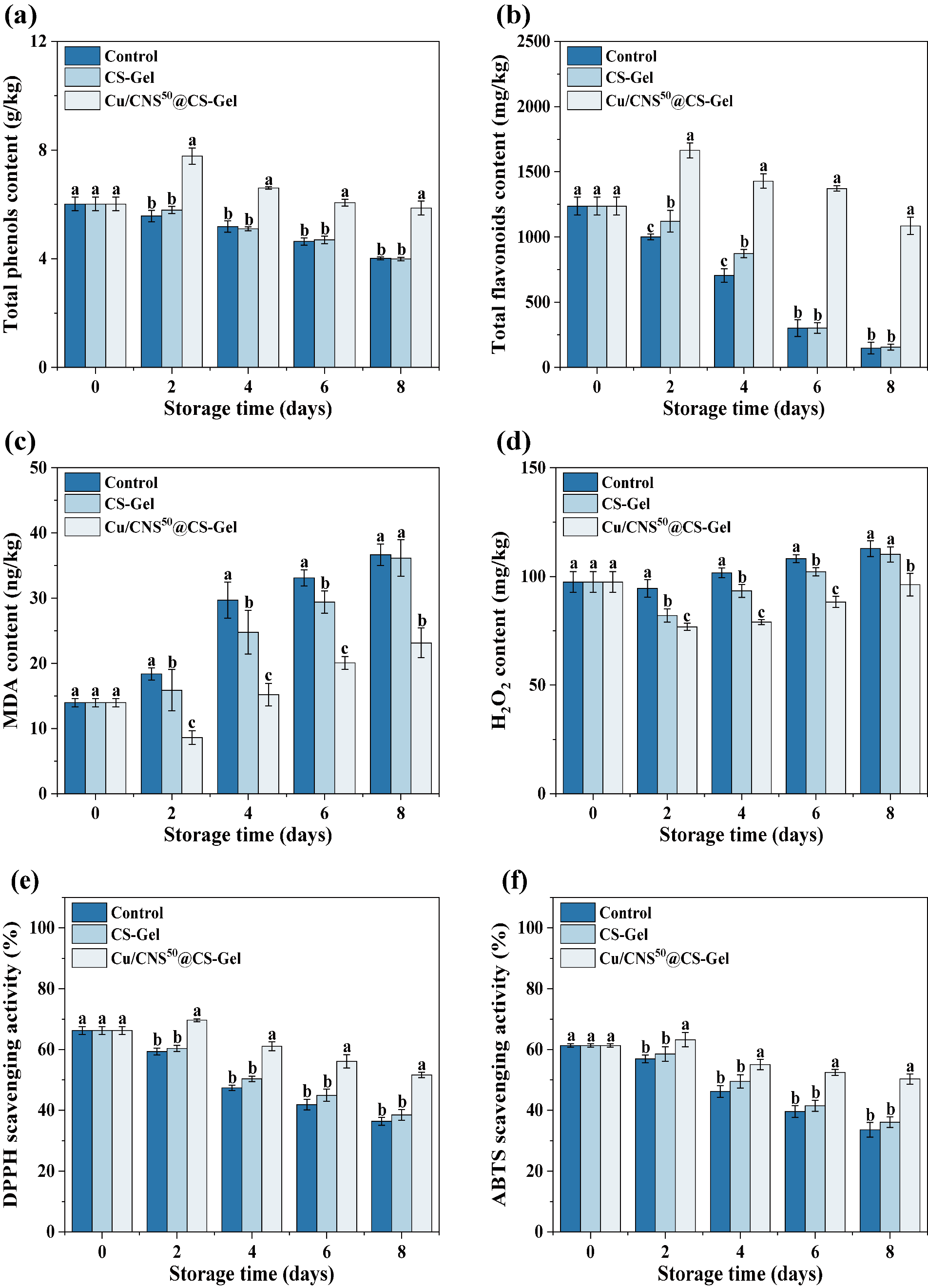


**Fig. S33.** Total phenols content (a), total flavonoids content (b), MDA content (c), H_2_O_2_ content (d), DPPH scavenging activity (e) and ABTS scavenging activity (f) of postharvest strawberries (non-climacteric fruit). Means followed by different letters are significantly different at *P* < 0.05.


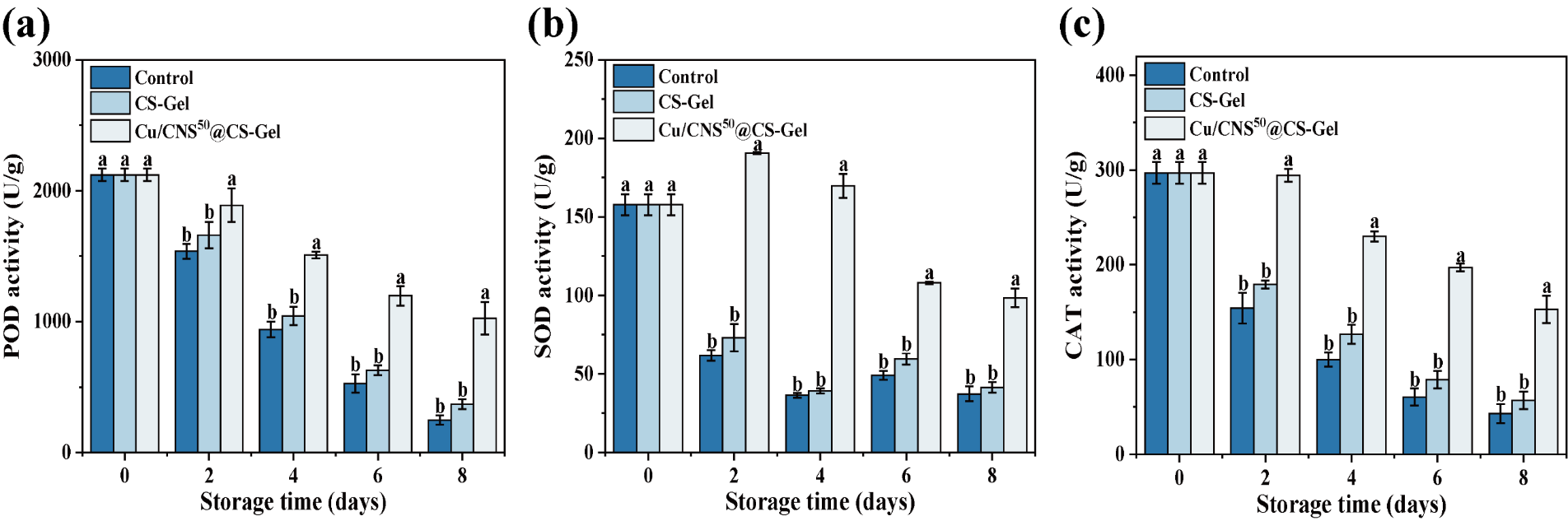


**Fig. S34.** POD (a), SOD (b) and CAT (c) activities of postharvest strawberries (non-climacteric fruit). Means followed by different letters are significantly different at *P* < 0.05.


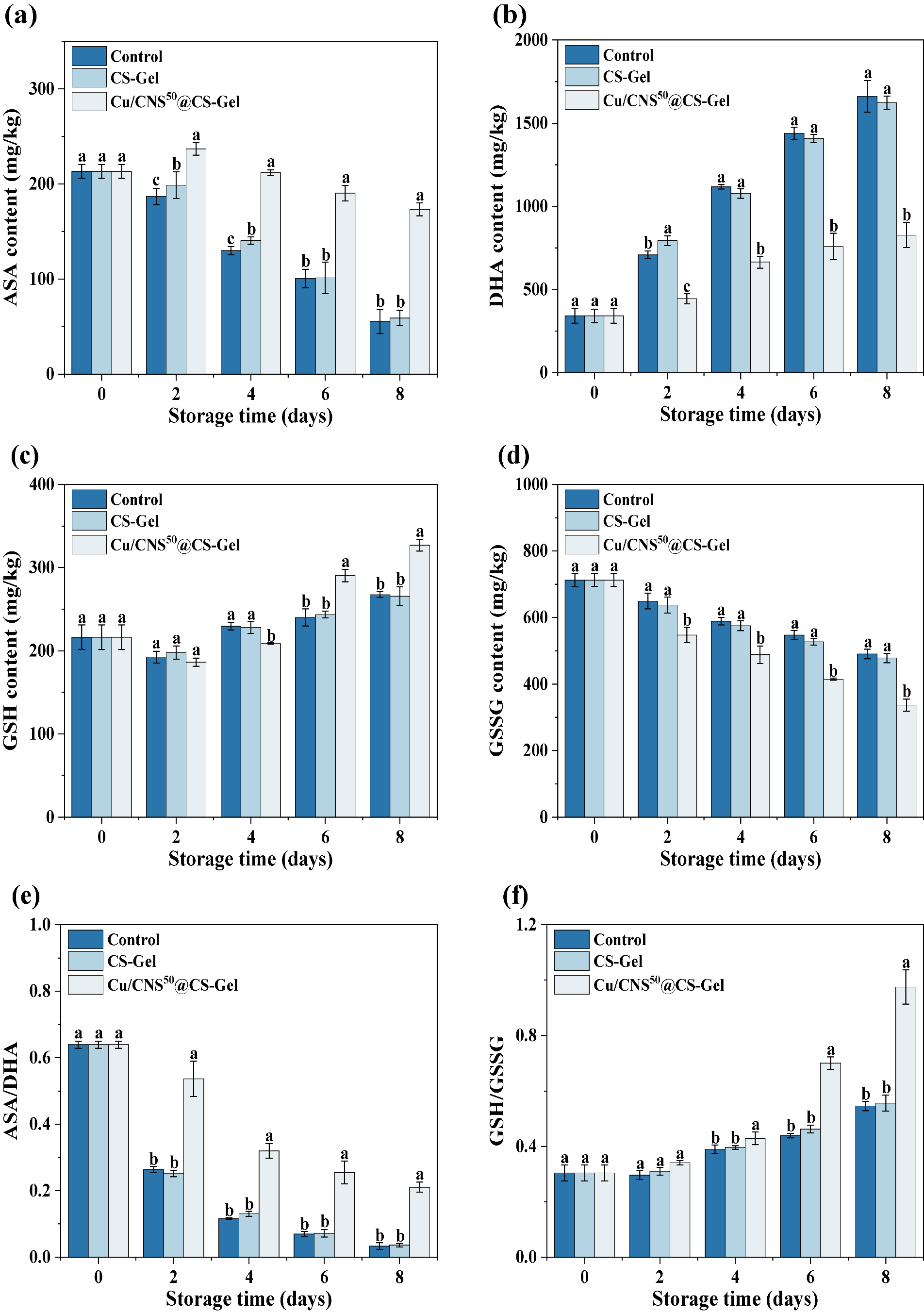


**Fig. S35.** ASA content (a), DHA content (b), GSH content (c), GSSG content (d), ASA/DHA (e) and GSH/GSSG (f) of postharvest strawberries (non-climacteric fruit). Means followed by different letters are significantly different at *P* < 0.05.


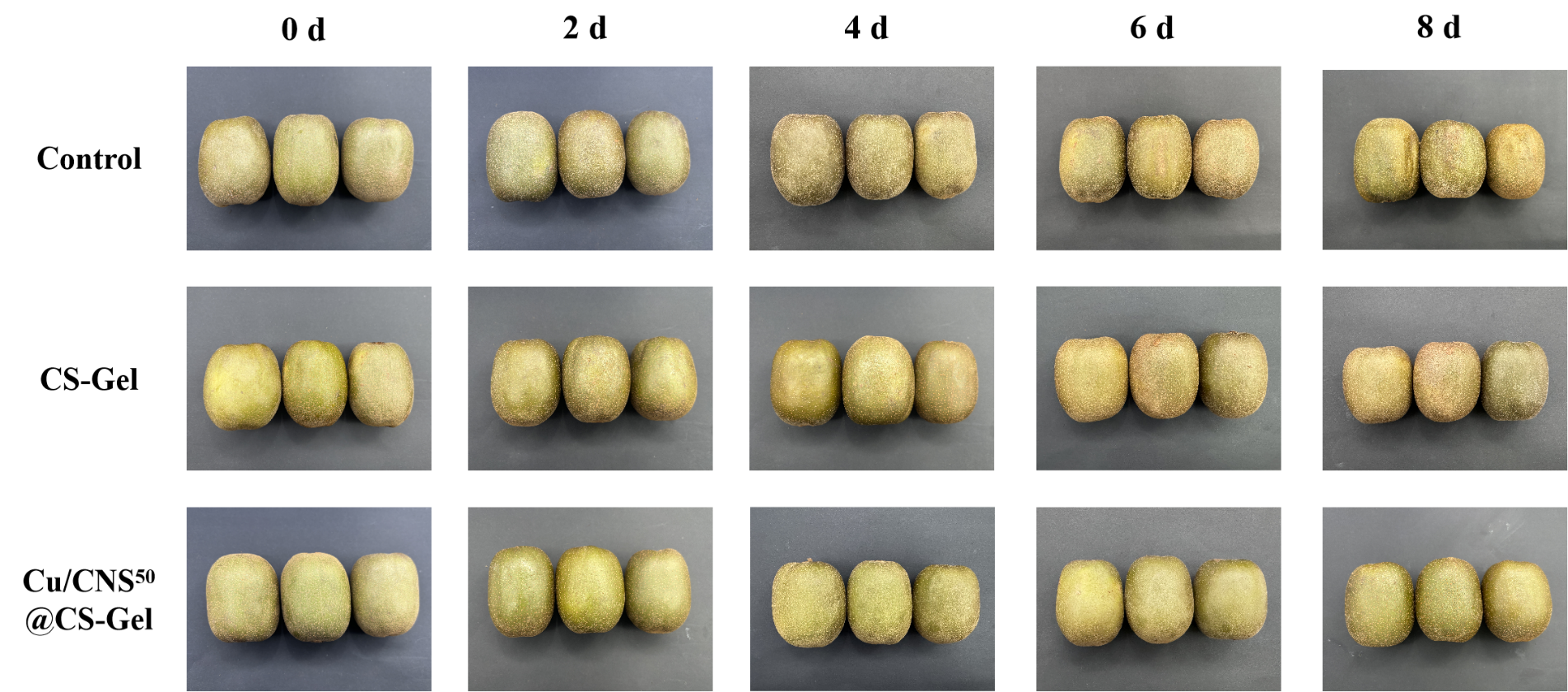


**Fig. S36.** Appearance pictures of postharvest kiwifruits (climacteric fruit).


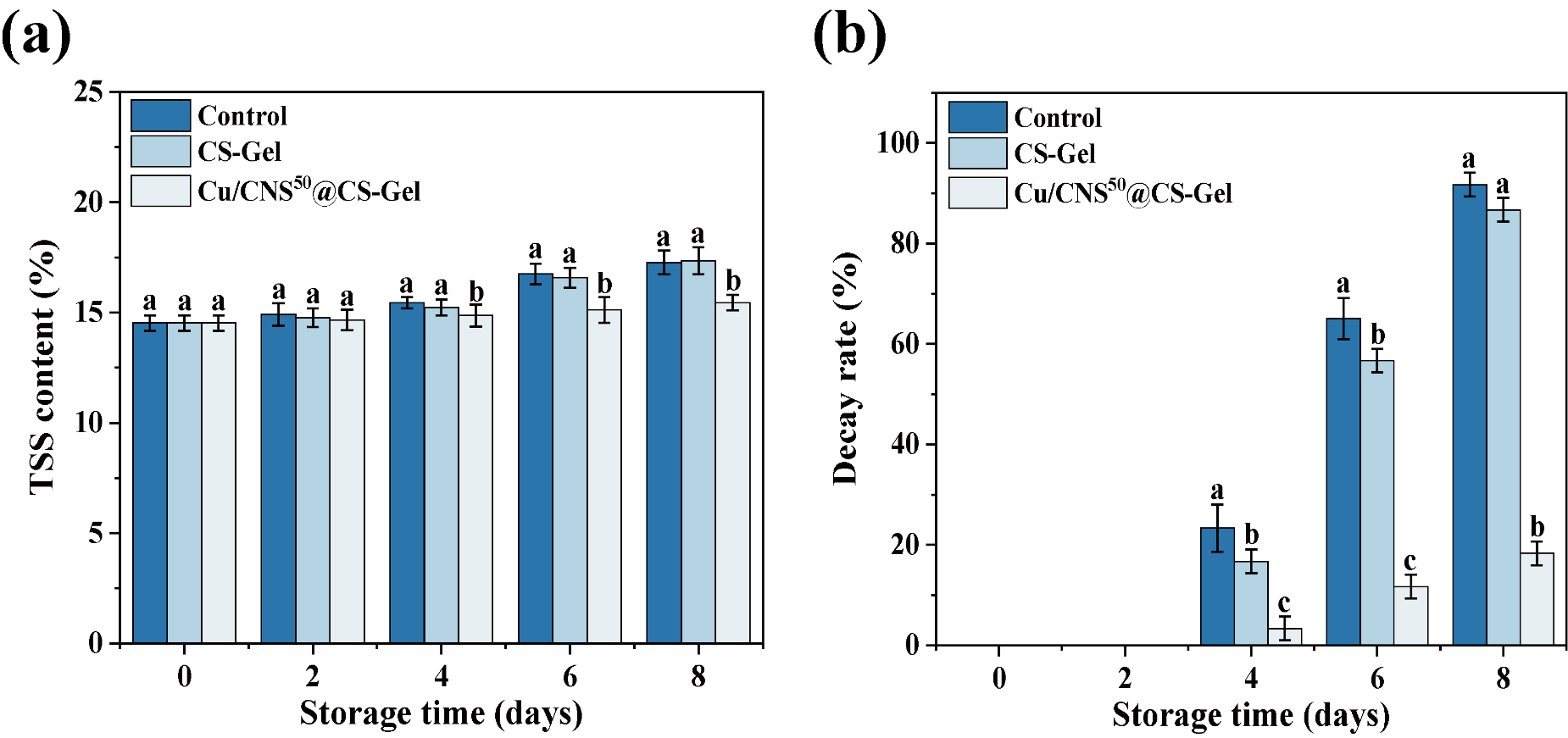


**Fig. S37.** TSS content (a), and decay rate (b) of postharvest kiwifruits (climacteric fruit). Means followed by different letters are significantly different at *P* < 0.05.


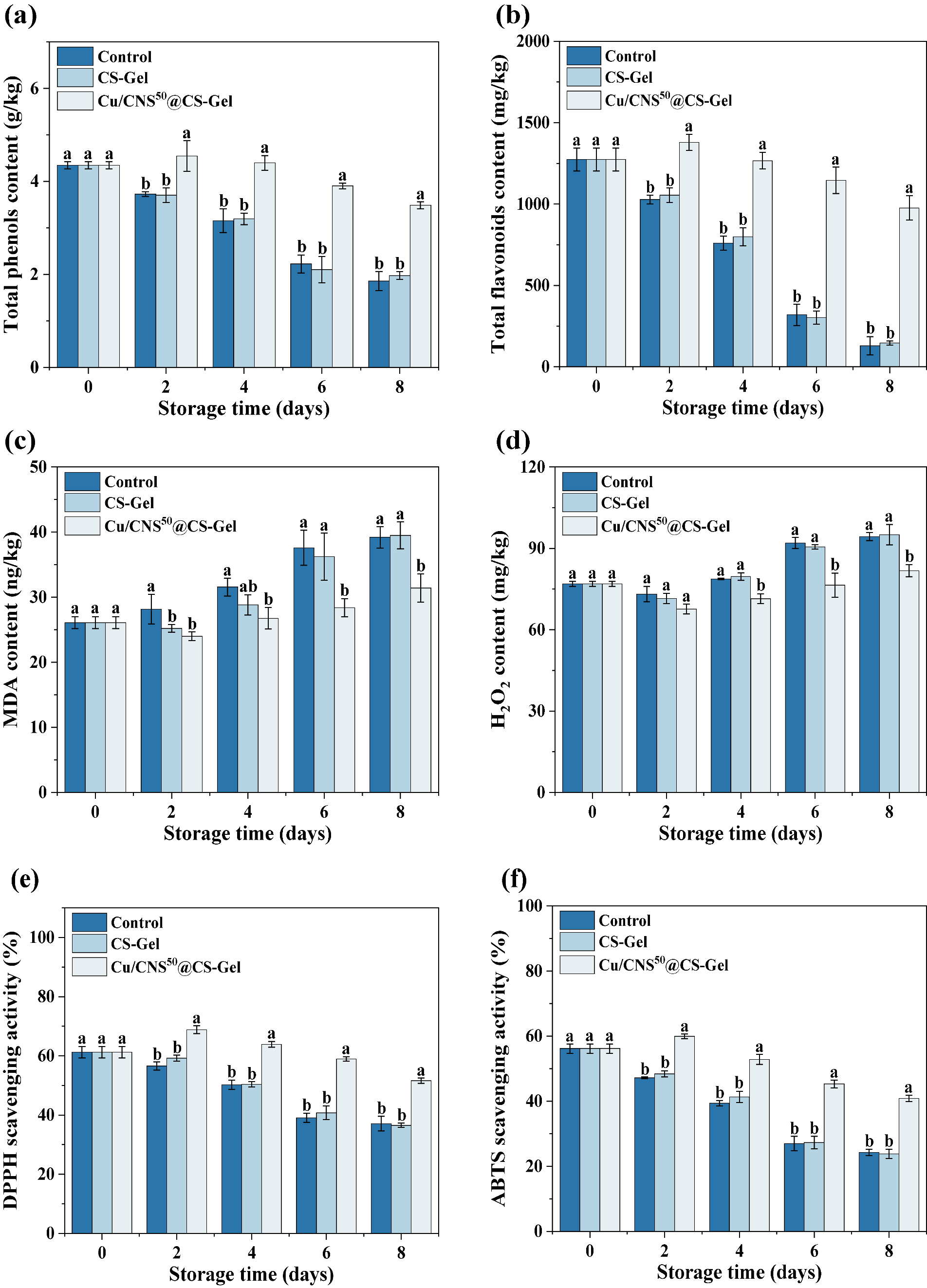


**Fig. S38.** Total phenols content (a), total flavonoids content (b), MDA content (c), H_2_O_2_ content (d), DPPH scavenging activity (e) and ABTS scavenging activity (f) of postharvest kiwifruits (climacteric fruit). Means followed by different letters are significantly different at *P* < 0.05.


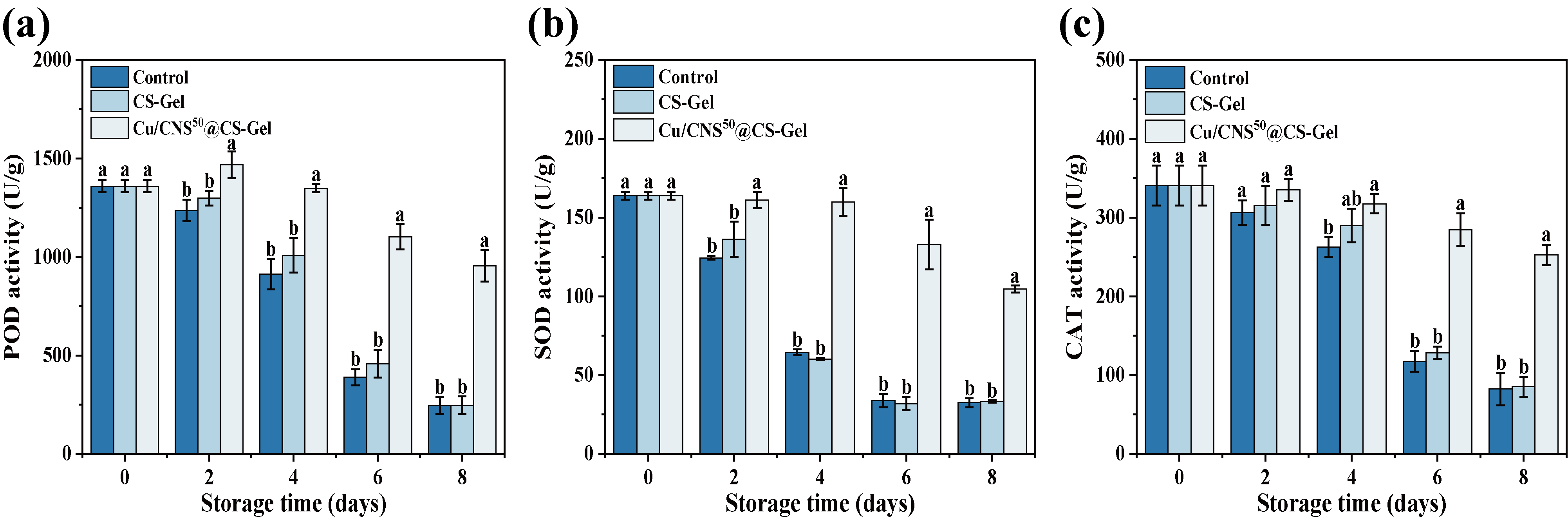


**Fig. S39.** POD (a), SOD (b) and CAT (c) activities of postharvest kiwifruits (climacteric fruit). Means followed by different letters are significantly different at *P* < 0.05.


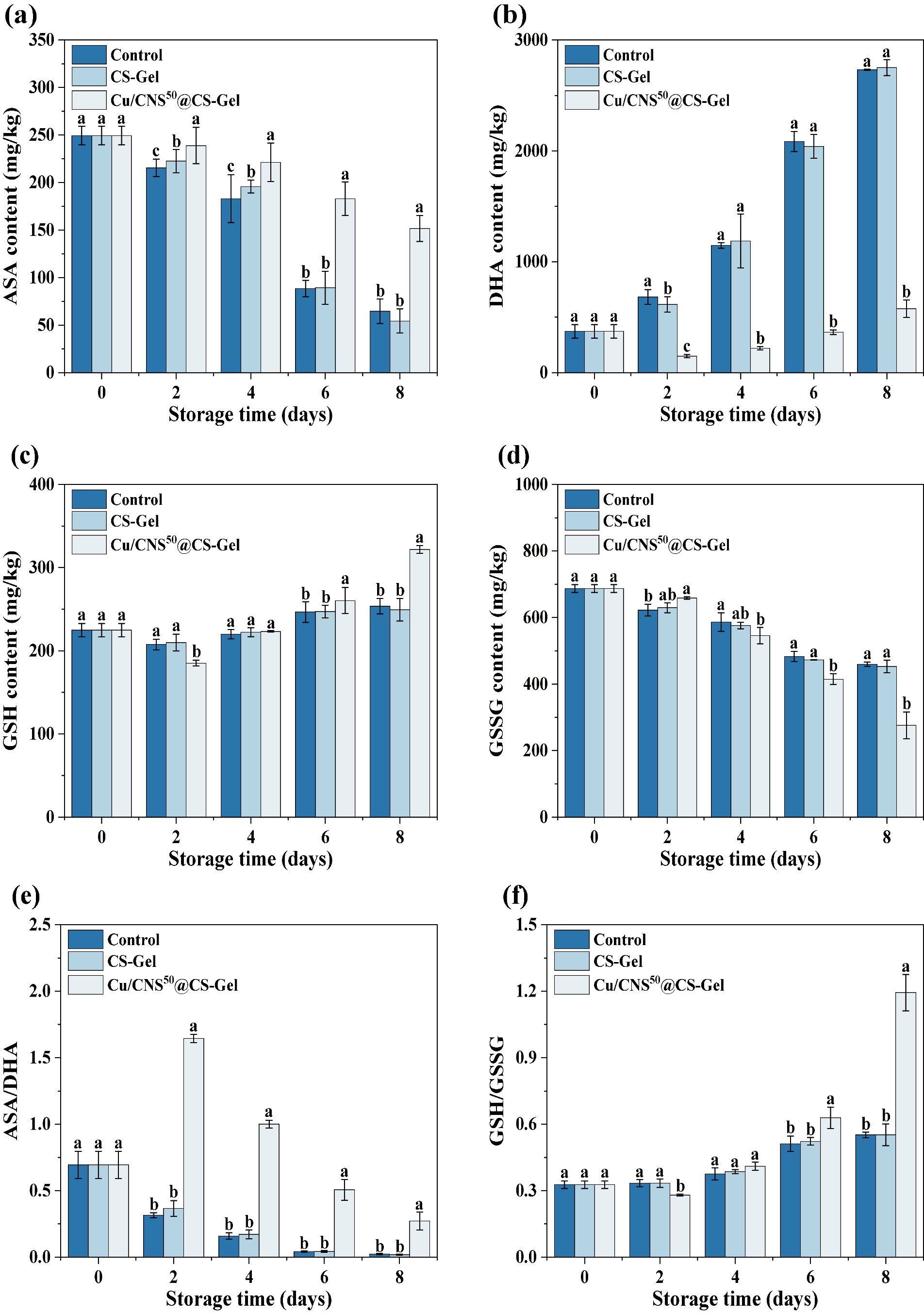


**Fig. S40.** ASA content (a), DHA content (b), GSH content (c), GSSG content (d), ASA/DHA (e) and GSH/GSSG (f) of postharvest kiwifruits (climacteric fruit). Means followed by different letters are significantly different at *P* < 0.05.


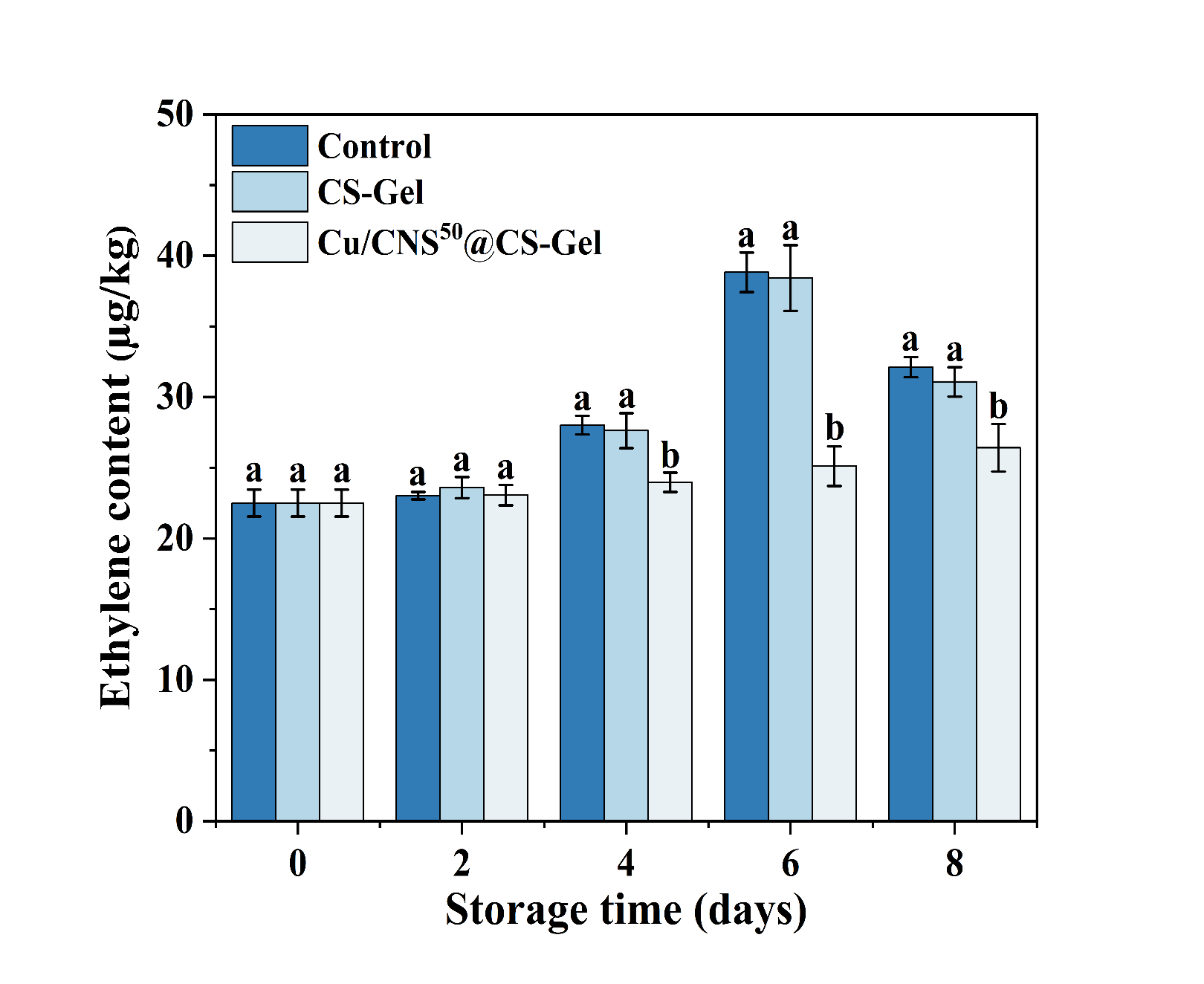


**Fig. S41.** Ethylene content of postharvest kiwifruits (climacteric fruit). Means followed by different letters are significantly different at *P* < 0.05.


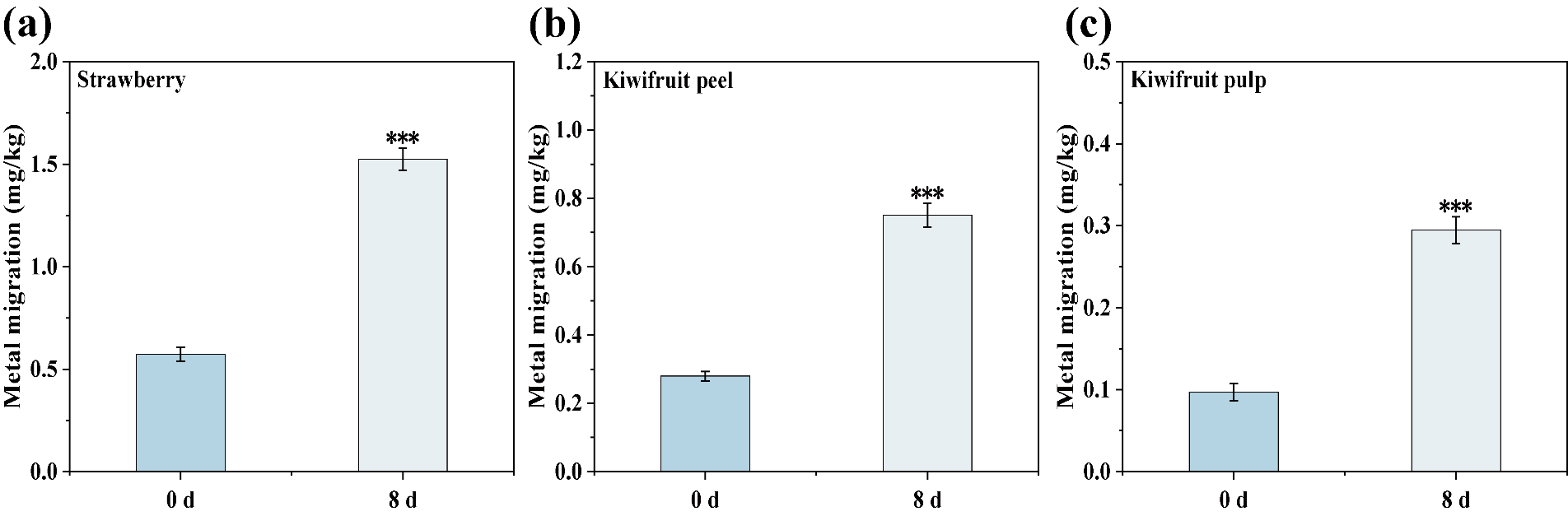


**Fig. S42.** Migration of Cu element from Cu/CNS^50^@CS-Gel film to strawberries (a), kiwifruits peel (b), and kiwifruits pulp (c) on days 0 and 8. The unpaired Student's bilateral t-test was used to assess the significance of the data: **P*<0.05, ***P*<0.01, ****P* <0.001.

**Table S1.** Raman data of D bond, G bond and I_D_/I_G_ for Cu/CN and Cu/CNS-1.

| **Samples** | **D bond (cm^-1^)** | **G bond (cm^-1^)** | **I_D_/I_G_** |
| --- | --- | --- | --- |
| Cu-CN | 1374.23 | 1590.09 | 1.07 |
| Cu/CNS-1 | 1370.13 | 1587.36 | 1.08 |

**Table S2.** The mass content of Cu and S element in Cu/CN, Cu/CNS-0.25, Cu/CNS-0.5 and Cu/CNS-1.

| **Sample** | **Test elements** | **Element content（g/kg)** | **Element content（%)** |  |
| --- | --- | --- | --- | --- |
| Cu-CN | Cu | 54.22 ± 0.28 | ~5.42 |  |
|  |  |  |  |  |
| Cu-CNS-0.25 | Cu | 50.68 ± 0.09 | ~5.07 |  |
|  |  |  |  |  |
| Cu-CNS-0.5 | Cu | 46.63 ± 0.11 | ~4.66 |  |
|  |  |  |  |  |
| Cu-CNS-1 | Cu | 42.66 ± 0.14 | ~4.27 |  |
|  |  |  |  |  |
| α-LA | S | 325.73 ± 1.17 | ~32.57 |  |
|  |  |  |  |  |
| Cu-CNS-0.25 | S | 21.02 ± 0.05 | ~2.10 |  |
|  |  |  |  |  |
| Cu-CNS-0.5 | S | 45.22 ± 0.51 | ~4.52 |  |
|  |  |  |  |  |
| Cu-CNS-1 | S | 69.20 ± 0.26 | ~6.92 |  |
|  |  |  |  |  |

**Table S3.** EXAFS fitting parameters at the Cu K-edge for various samples.

| **Sample** | **Shell** | **CN^a^** | **R(Å)^b^** | **σ^2^(Å^2^)^c^** | **ΔE_0_(eV)^d^** | **K-range/Å^-1^** | **R-range/Å** | **R factor** |
| --- | --- | --- | --- | --- | --- | --- | --- | --- |
| Cu foil | Cu-Cu | 12* | 2.54±0.01 | 0.0087±0.0005 | 3.6±0.3 | 3.0-12.0 | 1.0-3.0 | 0.0042 |
| CuPc | Cu-N | 4.0±0.6 | 1.94±0.01 | 0.0028±0.0015 | 7.5±1.2 | 3.0-13.0 | 1.2-2.0 | 0.0139 |
| CuO | Cu-O | 4.0±0.2 | 1.95±0.01 | 0.0036±0.0005 | 7.8±0.2 | 3.0-12.0 | 1.0-3.3 | 0.0015 |
|  | Cu-O | 2.1±0.3 | 2.73±0.01 |  |  |  |  |  |
|  | Cu-Cu | 2.4±0.3 | 2.85±0.01 | 0.0049±0.0007 | -5.7±0.6 |  |  |  |
|  | Cu-Cu | 2.3±0.3 | 3.39±0.01 |  |  |  |  |  |
| Cu/CNS-1 | Cu-N | 0.5±0.2 | 1.98±0.01 | 0.0076±0.0011 | 3.2±0.3 | 3.0-12.0 | 1.0-2.3 | 0.0024 |
|  | Cu-S | 3.0±0.3 | 2.27±0.01 |  |  |  |  |  |

*^a^CN*, coordination number; *^b^R*, the distance to the neighboring atom; *^c^σ*^2^, Debye-Waller factor , the Mean Square Relative Displacement (MSRD); *^d^ΔE*_0_, inner potential correction; *R* factor indicates the goodness of the fit. *S*0^2^ was fixed to 0.859, accourding to the experimental EXAFS fit of Cu foil by fixing *CN* as the known crystallographic value. * This value was fixed during EXAFS fitting, based on the known structure of Cu. Error bounds that characterize the structural parameters obtained by EXAFS spectroscopy were estimated as CN ± 20%; R ± 1%; σ2 ± 20%; ΔE0 ± 20%. A reasonable range of EXAFS fitting parameters: 0.700 < *Ѕ*_0_^2^ < 1.000; *CN >* 0; *σ*^2^ > 0 Å^2^; |Δ*E*_0_| < 15 eV; *R* factor < 0.02.

**Table S4.** Comparison of the kinetic constants of Cu/CN, Cu/CNS-0.25, Cu/CNS-0.5 and Cu/CNS-1.

| **Different catalysts** | **SA^a)^ [U/mg]** | **R^2^** | **K_m_^b)^ [mM]** | **V_max_^c)^ [μM/s]** | **R^2^** |
| --- | --- | --- | --- | --- | --- |
| Cu/CN | 1.46 × 10^−2^ | 0.99389 | 0.529 | 0.069 | 0.99717 |
| Cu/CNS-0.25 | 2.14 × 10^−2^ | 0.99621 | 0.462 | 0.099 | 0.99948 |
| Cu/CNS-0.5 | 3.18 × 10^−2^ | 0.99601 | 0.501 | 0.144 | 0.99798 |
| Cu/CNS-1 | 3.46 × 10^−2^ | 0.99843 | 0.460 | 0.162 | 0.99802 |

a) SA: the specific activity expressed in units per milligram (U/mg) Cu/CN, Cu/CNS-0.25, Cu/CNS-0.5 and Cu/CNS-1;

b) K_m_: the Michaelis constant;

c) V_max_: the maximal reaction velocity;

**Table S5.** Compare the mass content of Cu single atom in Cu/CNS-1 with the reported studies.

| **Different catalysts** | **Application fields** | **Mass contents** | **References** |  |
| --- | --- | --- | --- | --- |
| Cu/CNS-1 | Fruit preservation | 4.27 wt% | This work |  |
| Cu SAzyme | Photothermal therapy | 0.24 wt %. | Angew. Chem. Int. Ed., 2022, 61, e202209245. |  |
| Cu-NC | Tumor therapy | 0.34 wt% | Adv. Funct. Mater., 2023, 33, 2305625. |  |
| Cu SA-DOX@COD | Immunotherapy of Prostate Cancer | 0.507 wt% | Adv. Funct. Mater., 2024, 34, 2405265. |  |
| S-Cu-ISA/SNC | Oxygen reduction | 0.73 wt% | Nat. Commun., 2020, 11, 3049. |  |
| Cu SASs/NPC | Wound therapy | 1.15 wt% | Bioact. Mater., 2021, 6, 4389-4401. |  |
| Cu/CN | CO_2_ Reduction | 2.5 wt% | ACS Nano, 2020, 14, 8584-8593. |  |
| FePc@2D-Cu–N–C | Biomimetic O_2_ activation | 4.37 wt% | Nat. Commun., 2024, 15, 2239. |  |
| Cu-SA/SNC | Oxygen reduction | 4.5 wt% | Energy Environ. Sci., 2019, 12, 3508-3514. |  |
| MoOx-Cu-Cys-PVP | Tumor therapy | 10.10 wt % | J. Am. Chem. Soc., 2023, 145, 4279-4293. |  |
| Ti_3_(AlxCu1-x)C_2_ | Element replacement | 17.02 wt% | ACS Nano, 2019, 13. |  |
| Cu-SAN | Galactose detection | 20.0 wt% | Small, 2024, 20, 2405986. |  |

**Table S6.** The potential copper ion migration of strawberries (pulp) and kiwifruits (both peel and pulp) samples collected on day 0 of storage.

| **Sample (0 d)** | **Test elements** | **Control group (mg/kg)** | **Treatment group (mg/kg)** | **Migration content (mg/kg)** |
| --- | --- | --- | --- | --- |
| Strawberry | Cu | 0.85 | 1.42 | 0.57 |
| kiwifruit (peel) | Cu | 1.29 | 1.57 | 0.28 |
| kiwifruit (pulp) | Cu | 1.33 | 1.43 | 0.10 |

**Table S7.** The potential copper ion migration of strawberries (pulp) and kiwifruits (both peel and pulp) samples collected on day 8 of storage.

| **Sample (8 d)** | **Test elements** | **Control group (mg/kg)** | **Treatment group (mg/kg)** | **Migration content (mg/kg)** |
| --- | --- | --- | --- | --- |
| Strawberry | Cu | 1.27 | 2.79 | 1.52 |
| kiwifruit (peel) | Cu | 2.39 | 3.14 | 0.75 |
| kiwifruit (pulp) | Cu | 1.13 | 1.42 | 0.29 |
